# An ideographic study into physiology, alcohol craving and lapses during one hundred days of daily life monitoring

**DOI:** 10.1016/j.abrep.2022.100443

**Published:** 2022-06-26

**Authors:** Hendrika G. van Lier, Matthijs L. Noordzij, Marcel E. Pieterse, Marloes G. Postel, Miriam M.R. Vollenbroek-Hutten, Hein A. de Haan, Jan Maarten C. Schraagen

**Affiliations:** a Department of Cognition, Data and Education, University of Twente, Postbus 217, 7500 AE Enschede, the Netherlands; b Department of Psychology, Health and Technology, University of Twente, Postbus 217, 7500 AE Enschede, the Netherlands; c Research Group Technology, Health & Care, Saxion University of Applied Sciences, Enschede, the Netherlands; d Department of Biomedical Signals and Systems, Telemedicine Group, University of Twente, Postbus 217, 7500 AE Enschede, the Netherlands; e Medical Spectrum Twente, Enschede, the Netherlands; f Tactus Addiction Treatment, Postbus 154, 7400 AD Deventer, the Netherlands; g GGNet FPA De Boog, Vordenseweg 12, 7231 PA Warnsveld, the Netherlands; h Netherlands Organization for Applied Scientific Research (TNO), Soesterberg, the Netherlands

**Keywords:** Alcohol craving, Cardiovascular activity, Electrodermal activity, Ecological momentary assessment

## Abstract

**Introduction:**

Alcohol craving is a highly challenging obstacle to achieve long-term abstinence. Making alcohol use disorder patients timely aware of high-risk craving situations may protect them against relapse by prompting them to mobilize their coping resources. Current advances in wearable and smart-phone technology provide novel opportunities for the development of detecting these situations of heightened risk of craving, by enabling continuous tracking of fluctuations in psychological and physiological parameters. The present study therefore aims to determine the association between self-reported craving and relapses, and between heightened physiological activity. Specifically, we measured cardiovascular and electrodermal activity, and self-reported craving during one hundred days in the daily life of people trying to recover from alcoholism. The secondary aim is to study whether the association between physiology and craving can be strengthened by the inclusion of context related psychological parameters.

**Methods:**

An intensive repeated and continuous measures in naturalistic settings case-study design was employed. Ten participants were monitored with wearable bio-sensors and answered multiple questions every three hours on a smartphone app about craving, lapsing and multiple evidence based contextual variables. The association between physiology, craving and lapses was explored using Matthews correlation coefficients both with a current and 3 h lagged design. The contextual variables were included in a decision tree together with the physiological parameters to explore the added effect on the correlation of these contextual variables.

**Results:**

The association between lapses and craving was highly different across individuals, varying between a weak to a strong association. The association between cardiovascular activity and heightened self-reported craving was negligible to weak, however with a high specificity, meaning that most craving events were accompanied by increase heart rate. However, the association between electrodermal activity and craving was lower than with cardiovascular activity for most participants, both prior (lagged) and during craving. For two of the participants the association between physiology and craving improved by adding contextual variables, however, precision was too low.

**Conclusions:**

People differ strongly in their bodily reactions and psychological experiences during the first months of their addiction treatment. No individual in our study had unique one-to-one mappings between on the one hand physiological or psychological precursors, and on the other hand craving and (re)lapses. Therefore, detecting high risk craving situations with both physiological activity measured with wearables and psychological precursors to alert people specifically for an imminent (re)lapse, does not seem viable on the basis of the current results. We do see an added benefit of using physiology during treatment, as physiology can help start the conversation about possible high risk craving situations during that week. This would also help the counselor to gain added insights into the fluctuating states of the clients, and help to ameliorate the recall bias of clients. The present study showed the possibility and paved the way for future intensive longitudinal designs integrating both physiological, psychological and contextual factors during the challenging and lengthy recovery from addiction.

## Highlights

• In a 100 days intensive study considerable intra- and interindividual differences were established in physiology and psychological craving of people with alcohol use disorder.
• For one third of the people heightened heart rate was associated with high craving.
• For most participants that reported lapses, lapses cooccurred with craving in at least 92% of the time.
• During treatment ambulatory physiological data can support the detection and discussion of possible high risk craving situations in innovative and reliable ways.

## 1 Introduction

### 1.1 Problem description

Alcohol craving, the urge to use alcohol (Martinotti et al., 2013), is viewed as a highly challenging obstacle for recovery from dependency (Lowman, Hunt, Litten, & Drummond, 2000). Identifying challenging moments of craving, by finding immediate precursors of craving and constructs that are associated with craving, provides the opportunity to develop treatments to facilitate recovery. Making alcohol use disorder (AUD) patients in treatment timely aware of high-risk situations may prompt them to mobilize their coping resources (Rohsenow and Monti, 1999). After years of drinking, these AUD patients have become over-sensitized to such high-risk situations, evoking both physiological (Quintana et al., 2013) and psychological (Ooteman, Koeter, Verheul, Schippers, & Brink, 2006) responses, which may lead to lapsing. Lapses are defined as temporary drinking that violates the abstaining goal (Larimer et al., 1999). Unfortunately, many patients do not recognize such early responses in time and fail to take necessary precautions to prevent lapsing (Baker et al., 2004). Currently, little research has been performed outside the lab to identify these critical moments (Kuerbis et al., 2020, Witteman et al., 2015). Additionally, critical moments might be highly individualized (Drummond, 2001, Kavanagh et al., 2013). Current alcohol models are based on and tested with aggregated data, which do not allow to draw inference on how individuals experience craving over time (Fisher et al., 2018). Therefore, identifying individualized moments of high craving in a person’s natural environment is an important step to actively support a person to achieve long-term abstinence.

## 2 Theory

### 2.1 Craving and (Re)lapse

There is a debate about the definition and role of craving within alcohol addiction models (Alayan et al., 2018, Kavanagh et al., 2013). Most scholars (for a recent review see van Lier et al., 2018) posit a causal role for craving (Baker et al., 2004, Larimer et al., 1999, Reis, 2012, Robinson and Berridge, 1993, Tiffany and Conklin, 2000, Verheul et al., 1999), with a few notable exceptions (e.g. Cox & Klinger, 1988). Some studies find a significant relation between craving and relapse (DeMartini et al., 2020, Higley et al., 2011, Miller et al., 1996, Waters et al., 2020), whereas multiple other studies do not (Cooney et al., 1997, Holt et al., 2012, Krahn et al., 2005). Cooney et al. (1997) hypothesize that craving may only occur in a subset of patients, possibly explaining why the relation between craving and relapse is missing in some AUD patients and therefore low in cross-sectional studies. Currently, relapse is often defined as a dichotomized construct within a study, whether a participant does or does not relapse in the whole study in relation to the amount of craving reported. Finally, the definition of relapse in these studies is very broad and can differ from any amount of drinking to drinking large amounts in specific periods, for an overview see Maisto et al. (2016). Consequently, the relation between craving and actual relapse in empirical studies remains complicated (Waters et al., 2020).

Serre et al. (2015) argue that although craving is believed to play a major role in the process of (re)lapse, this association remains poorly understood because of the time-limited nature of craving and retrospective reporting bias present in many studies. Therefore, a significant next step would be to investigate the relationship between craving and lapses within person over a longer timeframe, making it possible to investigate how often individuals actually can or cannot resist different levels of craving in daily life.

### 2.2 Craving and physiology

If heightened craving is predictive of lapses within person, measuring craving continuously would clearly open novel avenues for preventing (re)lapsing. For example, helping patients to use their coping skills or call in help at critical moments of craving (Rohsenow & Monti, 1999). Currently, craving is most often measured subjectively using a questionnaire, interview (Cooney et al., 1997, DeMartini et al., 2020, Higley et al., 2011, Miller et al., 1996) or daily diary (Holt et al., 2012, Krahn et al., 2005, Waters et al., 2020). However, relying on self-reports in treatment or research for extensive and longer timeframes (i.e. more than two weeks) is burdensome and undesirable, due to retrospective bias (Shiffman, 2009). Therefore, Wray, Merrill and Monti (2014) propose to substitute self-reports with physiological measures. In particular, electrodermal activity (EDA) and cardiovascular activity (CVA) are frequently used physiological measures to investigate the relation with craving in a laboratory setting (Ooteman et al., 2006). Rosenberg (2009) showed that in a laboratory setting heightened physiological (heart rate and sweating) and self-reported craving responses do exist at the same time. Cardiovascular activity (CVA), indicates to what extent the autonomic nervous system is responding to changing situational demands (Appelhans & Luecken, 2006). Patients with substance related disorders might therefore benefit from direct (bio)feedback of CVA, as an early warning of relapse. In addition, EDA provides a measure mostly associated with the activity of the sympathetic nervous system. This parameter has been shown to allow differentiation, in a lab setting, between low- and high-risk individuals with respect to substance abuse (Taylor, 2004).

There are multiple perspectives on the role of physiology in addiction. In the past physiology was solely seen as a (conditioned) withdrawal symptom (Skinner & Aubin, 2010). More recently physiology has often been associated with cue-reactivity, a direct response after an alcohol stimulus (Reynolds and Monti, 2013, Witteman et al., 2015) in combination with a craving response. The idea of general response coherence, the co-occurrence of cognitive and physiological responses to certain cues or high risk situations, presumes (from the evolutionary point of view) that cognitive states prepare a person to take action when needed by their current surroundings, whether the surroundings pose a threat or an opportunity (Evers et al., 2014, Kuppens et al., 2010). However, Carter and Tiffany (1999) performed a *meta*-analysis on this general response coherence, where both a physiological and self-reported craving were measured after an alcohol cue, which only had a moderate correlation of 0.38 of physiology on self-reported craving. Since then, multiple explanations have been provided for the moderate relation between self-reported craving and physiology. Baker et al. (2004) suggested an individual might only experience craving if craving surpasses a certain threshold. Ooteman et al. (2006) argued that concordance of physiological cue reactivity and craving may only be present in a subgroup of alcoholic persons who are sensitive to their bodily reactions. Drummond (2001) hypothesized that craving has a more temporal character and that the interaction with physiology is probably too complex for the isolated laboratory based cues. In real life, stimuli or precursors to craving are less clear-cut, more personal, and therefore it is more difficult to determine the moment of the cue and its following response. In line with Ooteman et al., 2006, Kavanagh et al., 2013 proposed to measure craving at least daily and during situations of high risk for drinking or cravings’ impact on a person’s situation to better understand the relationship between cognitive craving and phsyiology.

In order to facilitate the temporal personalized characteristic of craving, longitudinal ecological momentary assessment (EMA; Shiffman, Stone, & Hufford, 2008) has been proposed. EMA methods are designed to address the personal differences between people and collect data on behavior, thoughts and feelings as they occur in the moment (Shiffman, 2009). Therefore, EMA includes the within person fluctuations and tackles the retrospective bias, in which participants are believed to make mistakes if they answer questions from memory (Shiffman, 2009). EMA type research allows researchers to draw conclusion on both the within-person fluctuations over time as well as between person differences (with a large enough sample) of craving (Drummond, 2001). However, alcohol craving EMA research is currently limited in scope, as mostly only cognitive and background variables are included. The physiological components of craving are currently only explored in lab studies. In a *meta*-analysis (Serre et al., 2015), 15 alcohol related EMA craving studies where included, none of the included parameters related to the physiological side of addiction. Therefore, insights on the temporal within-person relation between craving and physiology outside the lab are lacking.

### 2.3 Craving and influential contextual variables

EMA research takes place outside the lab, in the real world, where stimuli or precursors to craving or physiological responses are more complex (Drummond, 2001). In a literature review (van Lier et al., 2018), we explored potential evidence-based emotional, cognitive and contextual precursors of craving or relapse. This showed that negative affect, stress and social situations are relevant influential factors preceding craving (or relapse). This review is in agreement with EMA studies that found an effect of negative affect and positive social experiences on craving (Zheng et al., 2015). Additionally, the *meta*-analysis of Serre et al. (2015) found four EMA studies on the effect of negative affect on craving, of which three found a positive correlation. Furthermore, Shiffman, Paty, Gnys, Kassel and Hickcox (1996) described two factors that could further explain the influence of social situations as trigger setting of craving. They found that whether alcohol is available and whether alcohol is permitted in social situations are important contextual factors for alcohol craving in daily life. Cooney, Litt, Morse, Bauer, and Gaupp (1997) found that alcohol craving and nicotine craving are highly related in persons with both addictions. Finally, Rohsenow and Monti (1999) describe that craving is not directly related to relapse, but that this relation is mediated by own belief in effectiveness of coping skills.

### 2.4 Current study

Current advances in wearable and smart-phone technology provide novel opportunities for the detection of personalized situations with heightened risk of (re)lapsing, by enabling continuous tracking of fluctuations in psychological and physiological parameters (e.g., Intille, 2012). The current study explores the association between physiological measures and psychological (emotional and cognitive) craving and craving’s association with (re)lapse, by monitoring multiple individuals with alcohol addiction for a long period (100 days). In these hundred days the CVA and EDA of each participant were measured with a wearable device in combination with the collection of self-reported variables with a mobile device, including craving, (re)lapses, and contextual variables related to the social situation.

Summarizing, the primary aims of the present study were twofold: to determine (1) the within-person association between self-reported craving and relapses, and (2) the within-person association between heightened physiological activity and heightened self-reported craving during one hundred days of monitoring people trying to recover from AUD in daily life. The secondary aim is to study whether the association between physiology and craving is moderated by contextual variables.

## 3 Materials and methods

This study was an observational study with an Intensive Repeated and Continuous Measures in Naturalistic Settings Case-study design (Moskowitz et al., 2009). Participants were monitored with a wearable bio-sensor (E4 wristband of Empatica) and answered multiple questions every three hours on a smartphone app. This study was approved by the Medical Ethical Committee Twente (registration number: NL58392.044.16).

### 3.1 Participants

Participants were recruited from the AUD patients pool of the outpatient online (alcoholdebaas.nl) and face-to-face treatment of an addiction care facility in the Netherlands. All patients were assessed at the beginning of their treatment on the type and severity of substance use disorder by the Substance Abuse Module of the Composite International Diagnostic Interview (Compton et al., 1996). The recruitment of AUD patients started in September 2016 and continued to March 2017. Participants were asked to start participating once they set their main treatment goal (abstinence or less drinking), which was after approximately six weeks of treatment, since they were expected to start experiencing craving from then. We were interested in craving in the context of a clear abstinence goal. In this way drinking can be defined as a lapses. Additionally, without this goal it is likely that craving is less prominent and urgent than with this drinking goal. Without the explicit commitment to stop or severely cut back on drinking there is a much smaller psychological barrier, and associated craving, to drinking. Finally, lapses cannot be meaningfully defined without this abstinence goal. Prior to this treatment goal participants were likely to drink without much craving, since they were not trying to abstain from alcohol. Therefore, including participants prior to this six week mark was expected to lead to less or even no craving and unrepresentative drinking moments. Participants filled in an informed consent prior to inclusion in the study. Ten participants were included in the study, since one dropped out within a week due to difficulties with the use of the technology. Six men and four women participated in the study with an average age of 40 (sd = 11). The in- and exclusion criteria can be found in Appendix A.

### 3.2 Study design

**Fig. 1:**
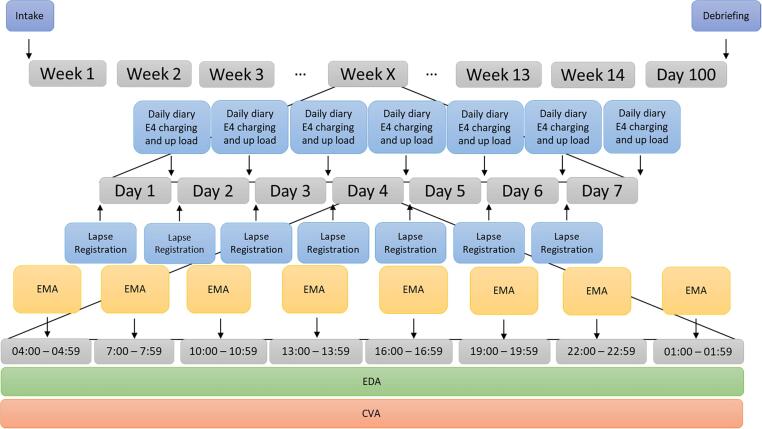
Overview of the complete study design of the 100 day experiment, first row is the complete study and the row beneath focuses on an example of a week and the row beneath on an example of a day in the experiment.

Participants carried their mobile phone throughout the day, which prompted them at set times (see Fig. 1) for assessments (time-contingent design). The time contingent design was found to be least burdensome for this set of questions in a pilot study with students (van Lier et al., 2017). At these set times the participants were asked to fill out some questions. These questions were discussed with four individuals diagnosed with AUD to reflect on their reactivity (Shiffman, 2009); specifically the possibility that the particular phrasing of the question itself induces additional craving. Further adjustments to the questions were made together with these experience experts. Additional questions were offered at the end of the day about their craving moments and alcohol use (daily diary). Administration with their mobile phone enhanced the ecological validity as the data were collected in real time and in their natural environment (Bolger & Laurenceau, 2013).

Next to the self-report questions, participants were asked to wear a biosensor wristband that measured electrodermal activity (EDA) and cardiovascular activity (CVA). Both measures are related to cognitive craving according to Carter en Tiffany (1999). Participants had to turn the E4 wristband on when waking up and download the data and charge the wristband during the night. The pilot study also showed (van Lier et al., 2017) that the usability of the wristband was high, but that participants wore the E4 from occasionally (few hours a day) to regularly (every day for five or more hours), therefore the micro incentives (further explained below) were added (Musthag et al., 2011).

The monitoring study lasted for 100 days, since the objective of our study is to define craving and lapses. In a review of 20 studies, Kirshenbaum et al. (2009) showed that there is a decelerating risk of 60% for relapse from initial abstinence (time point zero) to 100 days, and the hazard of relapse declines to nearly zero after 100 days. Therefore, one might conclude that any patient who has managed to achieve this milestone likely also possesses the ability to remain abstinent for one year or longer. Therefore, testing longer than 100 days will probably not add any new information. Fig. 1 shows the complete design of the study.

### 3.3 Apparatus

**Fig. 2:**
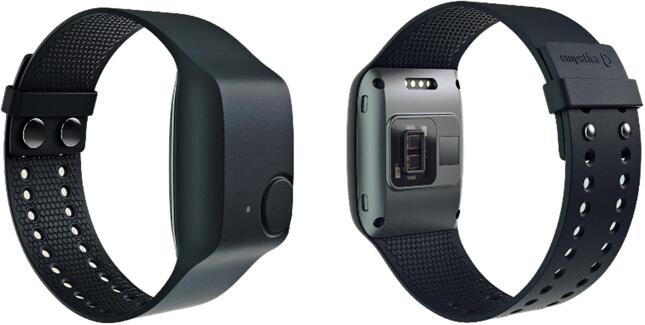
E4 wristband - Empatica, reprinted with permission.

The E4 wristband of Empatica (see Fig. 2) was used to collect physiological measures: electrodermal activity (EDA) and cardiac vascular activity (CVA). Additionally, acceleration was collected with the E4 wristband to correct the EDA and CVA data when necessary for movement. In a previous study (van Lier et al., 2020), we performed a validation experiment on the E4, comparing it to a Thought Technology sensor T7500M, acquired with a Procomp Infinity unit which is a measurement on the fingers for the EDA and wrist for CVA. This is often seen as the golden standard location for EDA measures. The comparison showed that the E4 wearable is valid for the parameters instantaneous heart rate and SD during a high stress event, and for total amplitude of skin conductance responses only when studying strong sustained stressors.

In a pilot study (Enewoldsen et al., 2016), we reviewed the quality of the E4 data by following eight participants for a week. Of the EDA data, 90% was artifact-free as shown through noise analysis (Taylor et al., 2015). Fluctuations showed that people have between 0 and 24 skin conductance responses per minute, which is in line with prior findings (Boucsein et al., 2012). The blood volume pulse (BVP) data from this pilot study consisted of around 20% unrealistic values plus artifacts which were detected through the visualization (the exact artifact free data could not be determined).

### 3.4 Procedure

In the start meeting, demographic variables were collected and in the concluding interview an evaluation of the experiment was held. As mentioned before, participants received a questionnaire at the beginning and end of the day and multiple questions during the day and one at the end of the week:

**Time-contingent design** Alcohol Ecological Momentary Assessment studies range from two to eight questionnaires a day (Collins et al., 1998; Cooney et al., 2009, Helzer et al., 2006, Holt et al., 2012, Krahn et al., 2005, Litt et al., 2009, Miranda et al., 2014, Ray, 2013, Tidey et al., 2008; Wang et al., 2014). However, most of these studies use four or five predetermined moments, together with an event-contingent design where participants are asked to administer craving moments whenever they occur. Four to five times a day, is in line with the *Handbook of Research Methods Studying Daily Living* (Mehl & Conner, 2012), claiming the range to be between 4 and 10, normative being 6. Shiffman (2009a) showed that AUD patients might have alternative rhythms and therefore it is best to monitor them by the power on of their phones. We will not assume that they will turn off their phone, but all data missing at night-time (or structurally during the day) will be categorized as ‘sleeping’. Therefore, the self-reported questions that were collected during the day were administered at 7, 10, 13, 16, 19, 22, 1 and 4 o’ clock. Participants had a period of one hour to respond to the questions. A cumulative micro incentive of max 1 euro a day was given for each finished questionnaire. Musthag, Raij, Ganesan, Kumar and Shiffman (2011) showed that micro incentive studies are low cost, while ensuring high compliance, good data quality, and lower retention issues. When a “normal” sleeping rhythm is used, participants were expected to answer 5 questionnaires a day (see Fig. 2).

To lower the burden (van Lier et al., 2017), some additional questions about craving moments were administered at the end of the day. The administered moments experiencing craving during the day higher than 2 (on a scale of 0–10) were reported back to the participant, who was then asked to provide additional information. A 4G scheme (Events, Thoughts, Feelings and Behaviour; Shoda & LeeTiernan, 2002) for every high craving event (>2) and social activities during the entire day were administered at the end of the day.

### 3.5 Measures

**Lapse** There were two moments at which the participants could indicate that they lapsed. The first registration cue occurred at the end of every day, where the activity after craving was questioned, and one of the options was drinking alcohol (thus a lapse). The second registration cue was in the morning; drinking moments of the prior day could be registered, since it might happen that a participant did not answer the question the previous day because of being in an intoxicated state or already sleeping. If a participant would answer that they had been drinking and the number of alcoholic units was asked for, the latter was not used in the study.

**Fig. 3:**
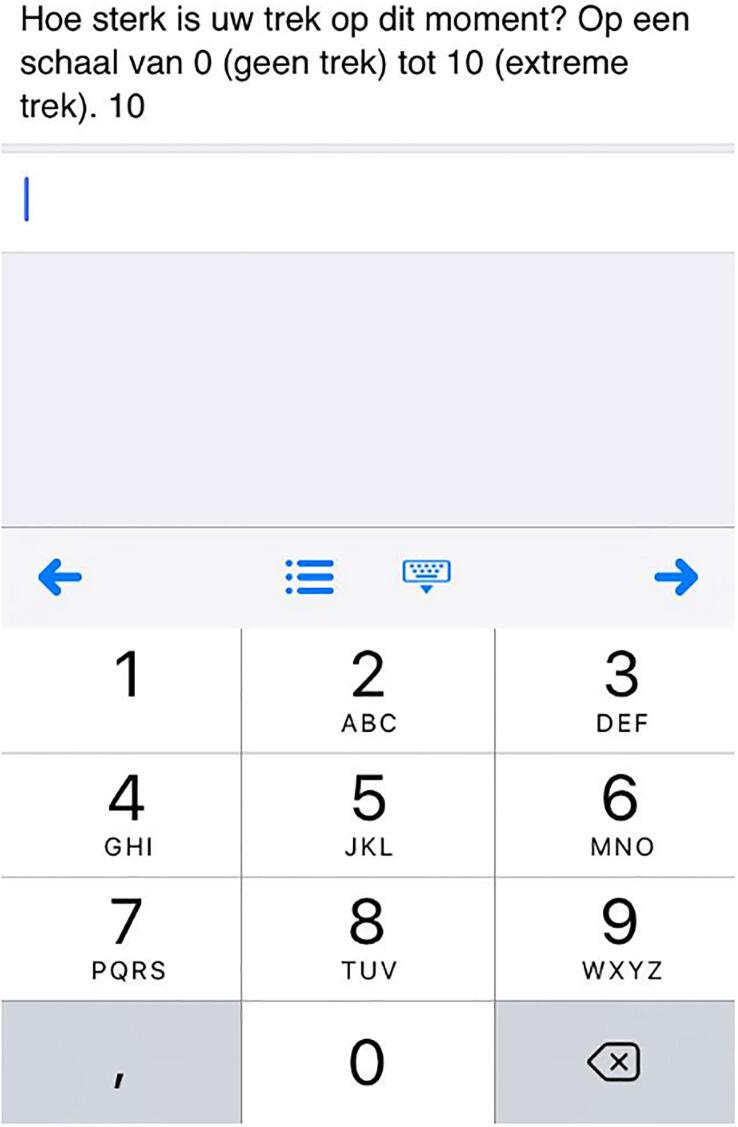
Print screen of the EMA app with craving question in Dutch.

**Self-reported craving**Ooteman, Koeter, Verheul, Schippers, and Brink (2006) argued that a single‐item measure is highly correlated with more extensive measures, where both are focused on the current state. Since the self-reported craving was administered every three hours, asking multiple craving items each time would increase the burden of this study unacceptably. Craving was measured with a 0–10 Likert scale, with 0 indicating no craving and 10 being high craving. Craving was measured by asking: “How strong is your craving currently?” (for the Dutch question see Fig. 3). Prior to the reactivity reflection session the question was: “How strong is your alcohol craving currently?” However the experience experts advocated to remove the use of the word alcohol in the questions due to their perception that this was likely to induce further craving.

**Total Amplitude - EDA** The E4 wristband uses electrodes to collect skin conductance (SC) to determine EDA measures. For the EDA data, Total amplitude was selected as parameter and mean HR for the CVA. Total Amplitude was selected since this is a combination of both the number or skin conductance responses and the amplitude, two often used measures of EDA data. Total Amplitude was retrieved from the SC through classical Trough-to-Peak analysis (TTP) (threshold for an SCR amplitude was set at 0.01 µS) (Boucsein, 2012). The data was analyzed with Ledalab, the default settings for filtering and smoothing from the program were used (Benedek & Kaernbach, 2010). The amplitude of a SC response was determined as the difference in conductance between response onset and response peak. The amplitudes were added in order to determine the total amplitude per minute. The total amplitude was therefore a function of both the number of SCRs and the amplitude of all these SCRs. The total amplitude per minute was then averaged over the tree hours prior to the end of possible question administration.

**Mean HR – CVA** The E4 wristband uses photoplethysmogram (PPG) to collect blood volume pulse (BVP) to determine CVA measures. Mean HR was selected since this parameter is familiar and a relevant indicator of CVA. The SD and RMSSD need higher quality data, that might not be guaranteed in the wild (Enewoldsen et al., 2016). Instantaneous HR can be determined by dividing the mean PP-interval per minute by 60 (seconds). HR was used instead of PP-interval, since HR is a better known transformation of the PP-interval. Mean HR per minute was again averaged over the three hours prior to the end of possible question administration.

**Movement** The E4 wristband has an onboard MEMS type 3-axis accelerometer that measures continuous gravitational force (g) applied to each of the three spatial dimensions (x, y, and z). The scale is limited to + -2g and the data is sampled at 32 HZ. Force was determined from the spatial data by calculating:

$$\mathrm{Force}=\sqrt{x^{2}+y^{2}+z^{2}}$$

From this force the standard deviation over the three hours prior to the end of possible question administration. Standard deviation was chosen over the mean, since the time intervals in this study are large. Mean movement is expected to be steady over hour, however SD of force incorporate these fluctuations.

**Other self-reported measures** Multiple other self-reported measures were collected, all as single item constructs with a 0 to 10 Likert scale (if not described otherwise). (1) Negative Affect, which was administered on a valence-arousal scale, a two-dimensional scale, with on the x-axis valence, from negative to positive and on the y-axis arousal, from low to high energy, (2) stress, (3) Social situations, which was divided in five possible social activities, namely “no social activity/work”, “friend/family”, “terrace/restaurant”, “party”, “other”. Other activities were hobby related or religious activities, (4) available, a yes or no question, (5) whether drinking was permitted, a yes or no question, (6) own belief in effectiveness of coping skills on a scale of 0 to 10 and (7) nicotine craving, also on a scale of 0 to 10.

### 3.6 Statistical analysis

#### 3.6.1 Descriptive statistics

The compliance rates, amount of craving above zero, number of lapses and the use of craving medication will be provided to give an overview of the data. Jones et al. (2018) found that the compliance rates were 70% on average in an alcohol dependent sample. If the compliance rates are lower than 80%, Stone and Shiffman (2002) warn for lack of representability of the true responses. Furthermore, the correlation between the compliance rate and lapses were computed to asses whether data might not be missing as random, where missing data could be expected to actually be more likely near lapse moments in alcohol dependent population (Stone and Shiffman, 2002).

#### 3.6.2 Confusion matrix

Cooney et al. (2009) showed that individuals suffering from alcohol addiction report craving in only 8% of the times they administered data, leading to unbalanced craving data. Imbalanced data are data where one classification is underrepresented (Ganganwar, 2012), in this case craving is often zero and less often above zero. As dichotomization is suitable to analyse imbalanced data (Luque et al., 2019), both physiological measures and self-reported craving were recoded into binary variables, whereas lapses were already binary. The data were dichotomized around a personal mean, meaning that above average craving is denoted 1 and below average craving is denoted 0. The cut-off point of using the personalized mean for the dichotomization was optimized over all participants for the Mathews correlation coefficient (MCC), where choosing a cut-off point higher or lower than the personalized mean would lead to either more false positives or false negatives. The MCC, sensitivity, specificity and precision are measures from a confusion matrix, and provide four metrics to analyse the performance of a dichotomized model. The MCC, sensitivity, specificity and precision will be determined for the three hours of physiology “during” answering the questionnaire (three hours prior to the end of the questionnaire registration) and “lagged”, meaning the three hours prior to that timeframe of self-reported craving. Other timeframes were also explored, namely one minute, 15 min, 30 min and one hour, but these resulted in lower or similar cross correlation with craving and will not be further reported.

The match between above average craving and above average physiology is called a True Positive, see Table 1, and a mismatch a False negative or False positive. A True Negative is when craving is reported below average and the measurement of physiology is also below average. Based on true and false positive, true and false negative from the confusion matrix four measures of coherence can be determined; the MCC, sensitivity, specificity and precision.

**Table 1:** Confusion matrix.

	**Mean HR/Total amplitude above average OR Relapse = 1**	**Mean HR/Total amplitude below average OR Relapse = 0**
**Self-reported Craving above average**	True Positives (TP)	False Positives (FP)
**Self-reported Craving below average**	False Negatives (FN)	True Negative (TN)

The MCC is a measure of the quality of two-class classifications (Chicco, 2017), according to the following formula:

(3) $$MCC=\frac{TP\;\;x\;TN\;-\;FP\;x\;FN\;}{\sqrt{\left(TP+FP)(TP+FN)(TN+FP)(TN+FN\right)}}$$

Sensitivity is the percentage of true positives correctly predicted to the total of predicted events of for example above average heart rate (Swift et al., 2020), according to the following formula:

(1) Sensitivity=TruePositives/(TruePositives+FalseNegatives)

Specificity is the percentage of true negatives predicted to the total of predicted events of for example above average heart rate (Swift et al., 2020), according to the following formula:

(2) Specificity=TrueNegatives/(TrueNegatives+FalsePositives)

Precision is the percentage of true positives predicted correctly to the total of the number of events of for example craving (Deng et al., 2016), according to the following formula:

(3) Precision=TruePositives/(TruePositives+FalsePositives)

The MCC’s outcomes are comparable to a correlation coefficient, with scores between −1 and +1. Where +1 represents a perfect predictive relation between physiology and craving, 0 indicates random prediction, and −1 a total disagreement between physiology and craving (when physiology is heightened, craving will be low and vice versa). The acceptable rate of the sensitivity and specificity differs per discipline and is dependent on the context. The precision gives some more information on the rate between the True Positives and False Positives, since the balance of the data has large implications of the outcome. Lalkhen and McCluskey (2008) note that 100% for both sensitivity and specificity is unrealistic in a practical context. For our study a low specificity would be especially problematic, where a low percentage of correct non-events, would mean that physiology is often below average when self-reported craving is actually above average. This would point towards two findings: First, heightened craving cannot be detected and possible high risk situations are therefore missed, meaning that physiology measured with wearable technology is not suitable for the detection of heightened craving. Second, if the wearable is believed to measure reliably, then the association between physiology and craving is questionable (for that person), since heightened craving does not occur with physiology. Concluding, both for the clinical relevance as empirical support for psycho-physiological concordance specificity should be high (above 80%; Marôco, 2018).

However, when specificity is high, low sensitivity might be less problematic, since this would only indicate that heightened craving is predicted more often for a person than relevant. This could have multiple plausible theoretical explanations. First, a person’s physiology responds to more triggers than just craving. EDA and CVA are both influenced by the sympathetic nervous system (SNS), responsible for the fight-or-flight response (Braithwaite, Watson, Jones, & Rowe, 2013) and this is triggered by multiple situations and not only by craving. Therefore, in the next section multiple contextual psychological variables, e.g. stressful events, are included. However, another influencer of CVA and EDA is movement. To assess whether a correction of movement should be included, movement was measured with the wearable and the association with mean HR and Total Amplitude was inspected. If certain outliers of mean HR, meaning above a certain threshold of movement, were strongly related to only movement and consistently not with craving, these outliers were to be removed. Second, as persons diagnosed with AUD have been found to be reluctant to admit their craving (Shiffman, 2009), or may even fail to recognize their own craving (Baker et al., 2004), physiology may detect certain cognitive responses that remain unconscious.

Similarly, the MCC, sensitivity, specificity and precision of self-reported craving and (re)lapse were determined. Here, the amount of expected false negatives will be bigger due to the lack or small amount of lapses expected per participants.

#### 3.6.3 Decision tree

Conditional inference trees (CI trees) were fit to this dichotomized data in order to explore which external factors, measured three hours prior, predicted the level (below average/above average) of self-reported craving. Thus, a prediction above or below average craving, was made with the lagged self-reported external factors and the current and lagged physiological parameters. Self-reported parameters were only included as lagged variables, since we are interested in the predictiveness of these parameters, in addition to physiological parameters, with craving. A CI tree is a decision tree algorithm for binary classifiers, which determines each split on permutation tests, attempting to differentiate between significant and insignificant improvements (see Sardá-Espinosa, Subbiaha, & Bartz-Beielstein, 2017 for a further explanation of CI trees in health data). The final tree was then built based on the entire dataset, without dividing in a training and test dataset, in order to use as much information as possible per participant and the results are aimed to be exploratory. The formula on which the tree was built is the following:

Craving* ∼ mean HR + mean HR^L^ + Total Amplitude + Total Amplitude^L^ + Stress^L^ + Coping^L^ + Valence^L^ + Arousal^L^ + Nicotine Craving^L^ + Social setting^L^ + Alcohol available^L^ + Permitted^L^ + friend/family^L^ + terrace/restaurant^L^ + party^L^,

where ^L^ are lagged variables.

## 4 Results

### 4.1 Descriptive statistics

In Table 2 the descriptive statistics of the participants’ self-reported craving and relapse data can be found.

**Table 2:** Descriptive statistics of the craving and relapse data. The first column shows the participant number, the second the number of answered questionnaires by an individual and the percentage of 500 possible administrations (5 a day for 100 days). The third is the number of above zero answers to the craving question, between brackets the percentage compared to the number of registration in the second column. The remaining columns indicate the number of lapses, mean and standard deviation of the reported craving levels (with a possible range of 0–10), and whether a participant used craving medication.

Participant	Compliance (500 registrations)	Alcohol Craving (>0)	No. Lapses	Mean Craving	SD Craving	Craving medication
1	**329** (66%)	**58** (18%)	16	0.81	1.85	Yes
2	**205** (41%)	**149** (73%)	0	2.87	2.89	No
3	**282** (56%)	**83** (29%)	8	1.63	2.83	Yes
4	**132** (26%)	**53** (40%)	0	1.58	2.54	No
5	**395** (79%)	**192** (49%)	28	1.12	1.85	No
6	**413** (82%)	**75** (18%)	26	1.22	2.87	No
7	**66** (13%)	**16** (25%)	0	0.56	1.49	No
8	**97** (19%)	**90** (93%)	19	3.87	2.33	Yes
9	**369** (74%)	**30** (8%)	0	0.42	1.48	No
10	**289** (58%)	**24** (8%)	6	0.40	1.49	No

#### 4.1.1 Compliance rates

Since we had round the clock measurements and participants were not expected to answer all questions, it is somewhat difficult to determine the exact compliance rate. When we assume a participant is awake for 16 h, he or she can theoretically answer on 6 time points at most. However, it is more realistic to assume five administration moments a day making the aimed for total compliance in a 100 days 500 registrations. Following this logic the compliance rates ranged from 13% to 82%, on average 66%. Stone and Shiffman (2002) warn for the representativeness of the sampling when the non-response is 20% or higher, especially when the data is expected to be not missing at random. However, this was a much longer study than what is typical (see for example; Van Berkel et al., 2017) and the compliance was difficult over this longer period. We included all participants that had a compliance rate of 40%, meaning at least 200 data points for an individual. Therefore, participants 4, 7 and 8 were excluded from the research. Additionally, there was a moderate positive correlation between compliance and relapse, in that the more a participant (re)lapsed the higher the compliance.

#### 4.1.2 Alcohol craving and lapses

We found differences in rates of experienced heightened alcohol craving of above 0, ranging from 8 to 73 percent (mean = 36%) of the administered data points. The mean craving was 1.45 (sd = 1.13), ranging from 0.4 to 3.87. Four persons registered not to experience a lapse at all. These participants did experience craving, but due to the absence of lapses they were excluded in this first analysis. These participants were included in further analyses into the relation between craving and physiology. Of the other participants, multiple lapses were registered, ranging from 6 to 28 lapses.

### 4.2 Confusion matrix

The confusion matrices was used to determine the association between (1) craving and lapses, and the association between (2) physiology, both HR and EDA, and craving.

#### 4.2.1 Association between craving and relapse

The MCC, sensitivity and specificity between craving and relapse during, prior to and 3 h during a (re)lapse of individuals who did relapse are presented in Table 3.

**Table 3:** MMC and Sensitivity/Specificity table of craving and lapses. The first column provides the participant number and the second and seventh shows how many were lapses compared to the total amount of registration where both craving was reported and a lapses was registered. The third and eight showed the MCC of craving prior and during a lapse. The fourth, fifth and nineth and tenth are the True Positive, the False Positive with the sensitivity and specificity between the brackets, the True Positive and False Positives add up to the total number of lapses. The sixth and eleventh column display the precision.

		High Craving during a Lapse					High Craving 3 h prior to a Lapse				
	Total no. Lapses (percentage of total administered data points)	No. Lapses with Craving data	MCC	TP (Sensitivity)	TN (Specificity**)**	Precision	No Lapses with Craving data 3 h prior	MCC	TP (Sensitivity)	TN (Specificity**)**	Precision
1	16 (2%)	12	0.19	7 (12%)	5 (98%)	58%	7	0.11	3 (5%)	4 (99%)	75%
3	8 (1%)	8	0.23	8 (8%)	0 (100%)	100%	5	0.18	5 (5%)	0 (100%)	100%
5	28 (2%)	19	0.21	12 (11%)	7 (98%)	63%	18	0.00	5 (4%)	13 (96%)	28%
6	26 (3%)	25	0.90	23 (25%)	2 (99.5%)	92%	22	0.00	5 (5%)	17 (95%)	22%
10	6 (1%)	5	0.44	5 (21%)	0 (100%)	100%	5	0.24	1 (4%)	4 (98%)	20%

**Fig. 4:**
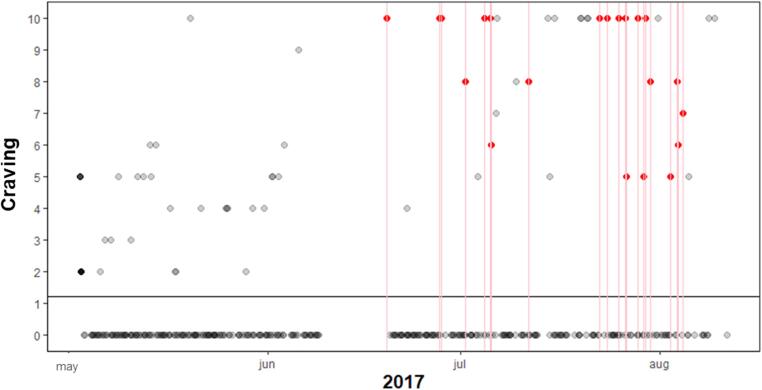
Participant 6 with high compliance, MCC and multiple lapses, red dots are craving measured during a lapse. The x-axis represents time, the y-axis craving. Craving ranges from 0 to 10, with 0 being low craving and 10 high. In the plot every (re)lapse is represented by a red line. The craving value during relapse are colored red, if not missing.

**Fig. 5:**
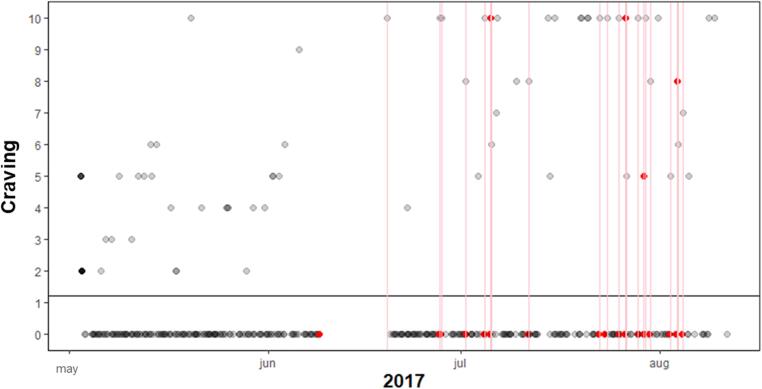
Participant 6 with high compliance, MCC and multiple relapses, red dots are craving measured 3 h prior to relapse. The x-axis represents time, the y-axis craving. Craving ranges from 0 to 10, with 0 being low craving and 10 high. In the plot every (re)lapse is represented by a red line. The craving value (3 h) prior to the lapse is colored red, if not missing. The black horizontal line is the mean craving.

The MCC for self-reported craving during relapse was between 0.19 and 0.90, indicating correlations varying between weak and very strong. In the 3 h prior to lapsing, the MCC was negligible to weak for all participants. Since there were so few relapse events in comparison to the complete data, the specificity for all participants was above 80%, but the sensitivity below 30%. However, three of the five participants had a precision of >92% of the lapses. Meaning, that craving (above average) did not always co-occur with lapses, however, if a lapse occurred, craving was almost always heightened at the same moment. For the craving prior to lapses this was the case for one or two persons (participant 3 and to some extent participant 1). This showed that craving in most individuals was not already heightened 3 h prior to a lapse. In Fig. 4, Fig. 5, two visualizations of the data of participant 6 are shown, the first with the association between lapses and concurrent craving and the latter between lapses and craving 3 h prior to these lapses. Participant 6 showed the highest MCC (0.90) with lapses during craving and the lowest (0.00) with craving prior to the lapses. Other participants have less clear patterns.

The figures show that participant 6 experienced nearly always heightened craving during lapses, however 3 h prior to these lapses craving was mostly absent, and only high in a few incidents. For the first lapse, only during the lapse craving was measured via self-report and not in the 3h prior.

#### 4.2.2 Association between mean HR and self-reported craving

The association between mean HR and self-reported craving was analysed by determining the MCC, sensitivity and specificity during craving and three hours prior to craving, and is shown in Table 4.

**Table 4:** MMC and Sensitivity/Specificity table of mean HR and craving. The first column provides the participant number and the second and seventh shows how many were above average HR measures compared to the total amount of registration where both craving was reported and HR was collected. The third and eight showed the MCC of HR prior and during craving. The fourth, fifth and ninth and tenth are the True Positive, the False Positive with the sensitivity and specificity between the brackets, the True Positive and False Positives add up to the total number of above average craving registrations. The sixth and eleventh column display the precision.

	Above average Mean HR during above average Craving					Above average Mean HR 3 h prior to above average Craving				
Partici-pant	Above average craving of the available data	MCC	TP (Sensitivity)	FP (Specificity)	Precision	Above average craving of the available data	MCC	TP (Sensitivity)	FP (Specificity)	Precision
1	42 of 208	0.25	26 (33%)	16 (88%)	62%	26 of 164	0.12	14 (21%)	12 (88%)	54%
2	14 of 32	0.27	10 (56%)	4 (71%)	71%	15 of 29	0.24	9 (64%)	6 (60%)	60%
3	26 of 107	0.29	24 (33%)	2 (94%)	92%	31 of 99	0.20	20 (41%)	11 (78%)	65%
5	57 of 289	0.25	42 (30%)	15 (90%)	74%	64 of 257	0.15	36 (33%)	28 (80%)	56%
6	63 of 228	0.02	29 (29%)	34 (73%)	46%	57 of 203	0.00	25 (28%)	32 (72%)	43%
9	23 of 113	0.26	18 (30%)	5 (91%)	78%	21 of 97	0.24	17 (30%)	4 (90%)	81%
10	15 of 73	-0.07	5 (17%)	10 (77%)	33%	13 of 64	-0.06	4 (17%)	9 (78%)	31%

**Fig. 6:**
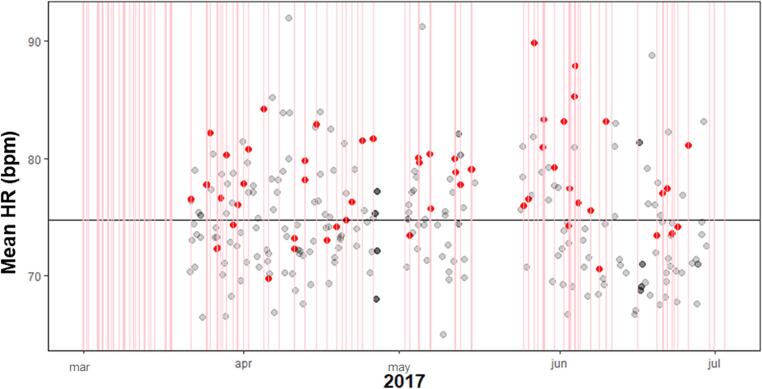
Participant 5 with specificity 90% and sensitivity 30% during above average craving. The red lines represent craving moments and the red dots the corresponding mean HR values. The black horizontal line is the average HR for the individual participant that was used to determine the sensitivity and specificity. The red dots above this mean line are True Positives and below this line are False Positives. The grey dots below the line indicate true negatives, and above the line false negatives. The dark grey dots are overlapping light grey dots.

**Fig. 7:**
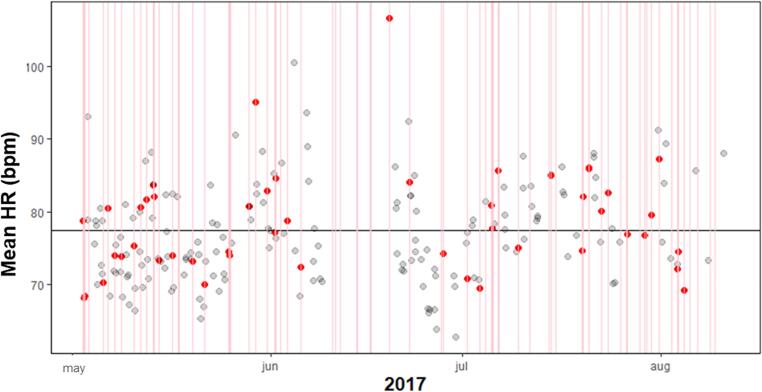
Participant 6 with specificity 73% and sensitivity 30% during above average craving. The red lines represent craving moments and the red dots the corresponding mean HR values. The black horizontal line is the average HR for the individual participant that was used to determine the sensitivity and specificity. The red dots above this mean line are True Positives and below this line are False Positives. The grey dots represent below the line indicating true negatives, and above the line false negatives.

All participants showed a negligible to weak MCC between HR and craving during and prior to craving. Four of the seven participants showed high specificity between 88% and 94% during craving, meaning that when their HR was below average, in only approximately 10% of the registrations they experienced above average craving. In these cases the sensitivity was 30% or higher. This showed that craving almost never occurred without heightened HR, but HR can be heightened in the absence of craving. This was also represented in the height of the precision, with precision rates of 71% or higher. Fig. 6 shows participant 5 who had high specificity and Fig. 7 participant 6 who had a lower specificity.

Three hours prior to the registration of craving the MCC, precision, specificity and sensitivity are lower for most participants, only two participants (2 and 9) have a specificity of 88 or higher. These participants have a moderate MCC and only participant 9 had a strong precision.  

**Movement**

The association of movement with mean HR and Craving showed no consistent outliers of mean HR related to movement (standard deviation of force). Therefore, no extra threshold based on the SD force could be included to increase the sensitivity of mean HR on craving. The details of this analysis can be found in Appendix B.

#### 4.2.3 Association between total amplitude and craving

For the coherence between Total Amplitude (TA) of the skin conductance responses identified in the EDA signal and self-reported craving a similar MCC, sensitivity and specificity table was made (see Table 5).

**Table 5:** MMC and Sensitivity/Specificity table of Total Amplitude and craving. The first column provides the participant number and the second and seventh show how many were above average for craving compared to the total amount of registration where both craving was reported and Total Amplitude was collected. The third and eight showed the MCC of TA prior and during craving. The fourth, fifth and nineth and tenth are the True Positive, the False Positive with the sensitivity and specificity between the brackets, the True Positive and False Positives add up to the total number of above average craving registrations. The sixth and eleventh column display the precision.

	Above average Total Amplitude 3 h prior to above average Craving					Above average Total Amplitude during above average Craving				
Partici-pant	Above average craving of the available data	MCC	TP (Sensitivity)	FP (Specificity)	Precision	Above average craving of the available data	MCC	TP (Sensitivity)	FP (Specificity)	Precision
1	39 of 217	-0.09	11 (14%)	28 (79%)	28%	50 of 269	0.00	16 (19%)	34 (81%)	32%
2	58 of 145	0.00	23 (40%)	35 (60%)	40%	58 of 157	-0.05	21 (34%)	37 (61%)	36%
3	0	–	–	–	–	0	–	–	–	–
5	73 of 294	0.01	23 (26%)	50 (75%)	31%	73 of 349	-0.01	18 (20%)	55 (79%)	25%
6	82 of 311	0.08	26 (33%)	56 (75%)	32%	82 of 383	0.09	27 (27%)	55 (81%)	33%
9	36 of 197	0.29	23 (33%)	13 (90%)	64%	45 of 249	0.32	28 (37%)	17 (90%)	62%
10	10 of 30	0.85	8 (100%)	2 (90%)	80%	9 of 30	0.84	8 (89%)	1 (95%)	89%

*Removal of outliers did not increase the sensitivity or specificity.

**Fig. 8:**
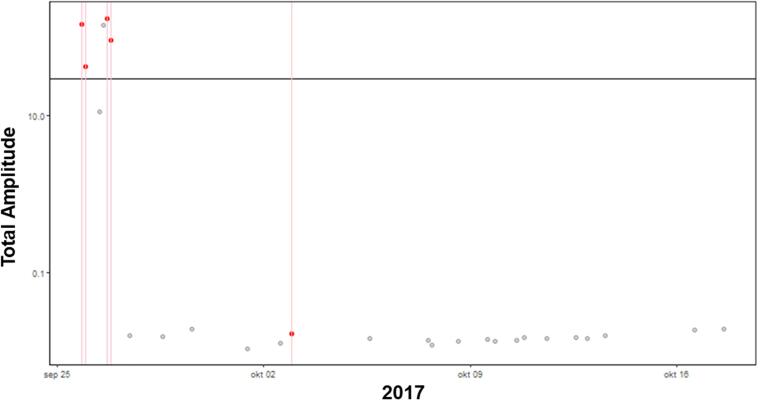
Participant 10 with a specificity of 89%, sensitivity of 84% and precision of 89% during above average craving. The red lines represent craving moments and the red dots the corresponding Total Amplitude values. The black horizontal line is the average Total Amplitude for the individual participant that was used to determine the sensitivity and specificity. The red dots above this mean line are True Positives and below this line are False Positives. The grey dots represent below the line indicating true negatives, and above the line false negatives.

**Fig. 9:**
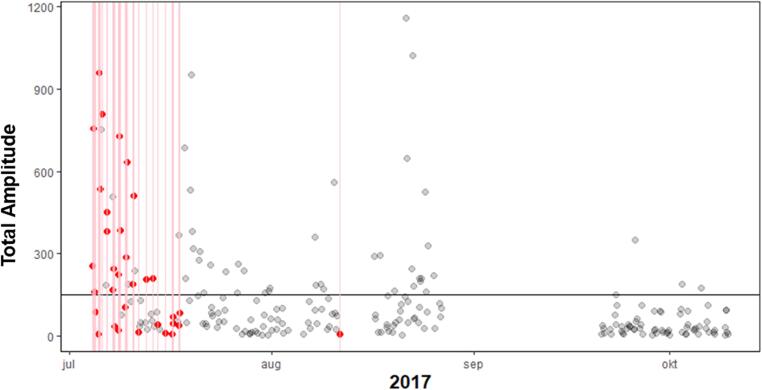
Participant 9 with a specificity of 90%, sensitivity of 37% and precision of 62% during above average craving. The red lines represent craving moments and the red dots the corresponding Total Amplitude values. The black horizontal line is the average Total Amplitude for the individual participant that was used to determine the sensitivity and specificity. The red dots above this mean line are True Positives and below this line are False Positives. The grey dots represent below the line indicating true negatives, and above the line false negatives.

Almost all participants showed a negligible to weak MCC between TA and craving during and prior to craving. Only participants 9 and 10 showed high specificity with TA for both during and 3 h prior an above average craving registration. Only for participant 10 (see Fig. 8) the MCC with EDA prior to craving was higher than during. It is important to note that participant 10 only had 30 entries where both craving and EDA were measured successfully. Participant 9 had many data entries, but with low sensitivity. The data of participant 9 is visualized in Fig. 9.

**Fig. 10:**
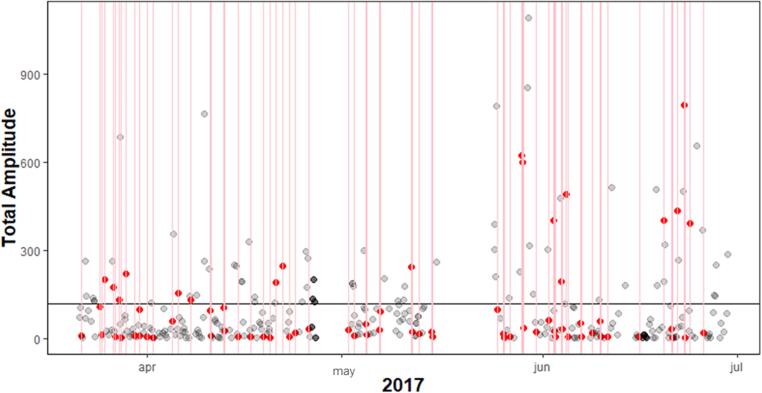
Participant 6 with a specificity of 81%, sensitivity of 27% and precision of 64% during above average craving. The red lines represent craving moments and the red dots the corresponding Total Amplitude values. The black horizontal line is the average Total Amplitude for the individual participant that was used to determine the sensitivity and specificity. The red dots above this mean line are True Positives and below this line are False Positives. The grey dots represent below the line indicating true negatives, and above the line false negatives.

Fig. 10 again show an example of a participant with low specificity.

**Movement**

The association of movement with Total Amplitude and Craving also showed no consistent outliers of Total Amplitude related to movement (standard deviation of force). Therefore, no extra threshold based on the SD force could be included to increase the sensitivity of Total Amplitude on craving. The details of this analysis can be found in Appendix C.

### 4.3 Decision tree

#### 4.3.1 Association between craving and both physiological and contextual variables

In Table 6 the results of the decision trees can be found where contextual variables are added in an attempt to improve the quality of the prediction of craving. Since we want to compare the MCC of the decision trees with the MCC’s found by using HR and TA, the MCC’s of HR and TA are given in the table in third and fourth column. The MCC during craving was given since this was almost always the highest MCC to compared to the MCC found 3 h prior to craving. Only for participant 10 the MCC with EDA prior to craving was higher than during (0.85 instead of 0.84).

**Table 6:** MCC and Sensitivity/specificity table of decision tree. The first column provides the participant number and the second the number of above average craving situations. The third and fourth column show the MCC for the HR and TA during craving. The fifth shows the MCC for the decision tree. The sixth and seventh are the True Positive and the False Positive with the sensitivity and specificity between the brackets, the True Positive and False Positives add up to the total number of above average craving registrations. The eight shows the MCC and last the significant features from the CI decision tree.

Participant	Total above average craving	MCC HR (during craving)	MCC TA (during craving)	MCC	True positives (Sensitivity)	False Positives (Specificity)	Precision	Sig features
1	58	0.25	0.00	0.22	11 (47%)	47 (85%)	19%	Coping^L^ → Stress^L^ Coping^L^ → HR^L^
2	84	0.27	-0.05	–	–	–	–	–
3	101	0.29	–	–	–	–	–	–
5	112	0.25	-0.01	–	–	–	–	–
6	91	0.02	0.09	0.31	15 (81%)	76 (82%)	16%	Stress^L^ → TA^L^ Stress^L^ → Arousal^L^ → Available^L^
9	52	0.26	0.32	0.53	18 (90%)	34 (92%)	35%	Stress^L^ → TA^L^ → TA Stress^L^ → Stress^L^ → Valence^L^
10	23	-0.07	0.84	–	–	–	–	–

For most participants a decision tree could not be found, meaning that no node could improve the prediction of craving compared to the null model (no craving). A null model is the largest class model, which is predicting there was no craving. The MCC of the decision tree was lower than the MCC with craving and physiology (both HR and EDA) for most of the participants, except two (participant 6 and 9). For participant 6 and 9 the MCC was higher than for HR or TA separately, for all other participants the MCC declined after including contextual variables. Furthermore, participants 6 and 9 had stress and total amplitude (lagged) as part of their decision tree. However, the precision for both was very low. Both had more craving incidents missed than found by their decision tree. Multiple participants have a specificity of above 80% and a sensitivity of above 30%. However, since the data is unbalanced, being overrepresented by low craving moments, often more above craving incidents are missed than registered (low precision).

## 5 Discussion

The primary aim of the present ideographic study is twofold: determining (1) the within-person association between self-reported craving and relapses, and (2) the within-person association between heightened physiological activity and heightened self-reported craving during one hundred days of monitoring people trying to recover from AUD in daily life. The secondary aim is to study whether the association between physiology and craving is moderated by contextual variables.

The association between (re)lapses and self-reported craving measured at a similar moment is strong for two of the five participants who relapsed and the other three participants had negligible to weak associations, prior and during relapse. HR has a negligible to weak association with concurrently measured heightened self-reported craving, and for HR three hours prior to craving all participants’ correlation’s are smaller. The association with EDA is lower than with HR for most participants, except for one participant, both prior and during craving. The association between physiology (both HR and EDA) and craving improves for two of the seven participants adding contextual variables, stress being the most consistent contributing factor, however the precision is low. Below, we will further discuss the results and their theoretical and practical implications for craving research and alcohol addiction treatment.

### 5.1 Association between craving and relapse

The association between (re)lapses and self-reported craving is evaluated to determine whether heightened self-reported craving would lead to (re)lapses for individuals. Of the 10 participants, six persons register experiencing lapses, which is in agreement with the findings of Kirshenbaum et al., (2009), who found that 60% of people in treatment relapses in the first 100 days. For the five participants who experienced lapses (one is excluded due to lack of other data), the association varies between weak (0.19) and strong (0.53). The overall finding is that the association between lapses and craving is highly different but consistently found across individuals, between weak to strong. The association between lapses and lagged craving is weaker. Two participants (40% of the lapses sample) showed consistent craving three hours prior to relapse. This moderate relation of craving during lapses is in line with prior cross-sectional non-EMA studies that found a relation between craving and relapse (Higley et al., 2011, Miller et al., 1996), given that this found cross-sectional relation translates to the individual level. However, it seems to be in contrast to prior EMA studies that found no between person association between craving and relapse (Cooney et al., 1997, Holt et al., 2012, Krahn et al., 2005). To the best of authors knowledge, this is the first study that researched the within-person association of craving with separate lapses or drinking moments in a longitudinal design, therefore comparison with between persons relapse studies is difficult.

The association between self-reported craving during lapses is further explored by the specificity and sensitivity. We find the overall specificity to be high, which indicates that almost all lapses are accompanied by heightened craving. However, two participants experienced approximately 40% of the lapses without craving. Whereas for the other participants this is as low as 0% to 8%, meaning craving is almost always experienced during a lapse. Again, this points to substantial heterogeneity within this population. This might explain the complex relation between craving and drinking, where not every person experiences craving prior to drinking. Cooney et al. (1997) argued that craving may only occur in a subset of individuals, this hypothesis seems less likely since all participants did experience some level of craving. Alternatively, in the cognitive processing model, Tiffany (1999) hypothesised that craving is only experienced when there is a blockage in the drinking process. Meaning that if a person drinks routinely, drinking can happen without craving. Two participant lapsed without craving, the other individuals experienced almost always craving during lapses. Tiffany’s cognitive processing model (1999) would imply that there first was some kind of blockage for these individuals (for example trying to stay abstinent or a bar being closed). Yet, the addition of the availability and allowance of alcohol and even own coping skills as a contextual predictors did not improve the association between physiology and craving within the individuals in our study.

The sensitivity of lapse prediction lies between 8% and 25%, showing that a participant lapsed at most a quarter of the times (s)he experienced craving. This shows that even people who lapse are mostly successful in abstaining. This might be explained by Rohsenow and Monti's (1999) proposal that craving and drinking are moderated by a person’s coping skills. A person can withstand craving and decide not to drink. Both Cooper et al., 1992, Cox and Klinger, 1988 advocate this idea that craving is mediated by motivation and a decision whether or not to drink.

### 5.2 Association between physiology and craving

The within person associations between physiology and self-reported craving are lower than the 0.38 between person correlation found by the *meta*-analysis of lab studies of Carter and Tiffany (1999). Of the participants five out of nine show a weak association between craving and HR between 0.25 and 0.29, the other four participants had a negligible association. This seems to be in line with Ooteman et al. (2006) who argued that concordance of physiological cue reactivity and craving may only be present in a subgroup of alcoholic persons. Their hypothesis is that certain individuals are more sensitive to their bodily reactions than other participants. However, even in this subgroup the associations are weak. The correlation is further explored by dividing it in sensitivity and specificity. All five participants had relatively high specificity (on average 90%) for the associations HR and Craving, meaning that not many craving “events” are not accompanied by above average HR. The low correlation is more explained by the low sensitivity of heightened HR. The low sensitivity indicates that no craving is present during a considerable number of moments of heightened HR. At these moments many other external or internal events may cause heightened HR (e.g., aggressive dog or excessive rumination about a previous argument), which are not all included in this research. Cacioppo et al. (2016) describes this a one-to-many psychophysiological mapping instead of an one-to-one relation between physiology and craving. Heightened HR maps on multiple psychological responses and not only on craving.

The associations between heightened self-reported craving and EDA is present in one participant. This participant shows nearly only increased TA during craving and vice versa. However, the overlay in data between EDA and craving is limited for this participant. This result is promising, but should be replicated in other individuals with more dense longitudinal data prior to drawing conclusions. Other participants have low precision, meaning that heightened craving are more often missed by TA than discovered. This finding is in contrast to Rosenberg (2009) who argues that there is a relation with both HR and EDA with respect to craving.

### 5.3 Association between (lagged) context precursors of craving

The secondary aim is to study whether the association of craving as obtained with physiological parameters, can be improved by the inclusion of context related variables such as stress, social activities (e.g. upcoming parties), and perceived self-control. The results of this study show that associations cannot be improved consistently across all individuals by including these evidence-based predictors of craving and relapse. This is in contrast to prior daily life studies (Serre et al., 2015) and our literature review (van Lier et al., 2018) that showed multiple context variables that were related to craving. The current study possibly differs from prior studies, since the current study investigates only the lagged influence of the context variables. Therefore, these contextual variables might have an effect more proximal to the moment of craving or longer than 3 h prior to craving, rather than 3 h prior to craving.

### 5.4 Clinical implications

This study shows that individuals experiencing relapse during their attempt to abstain from drinking, drank in 8% to 25% of craving incidents during 100 days. Conversely, 92% to 75% of craving incidents can be successfully resisted by these participants. This suggests that in at least a part of people undergoing alcohol dependence treatment, preventing relapse comes down to helping them to get through those few critical events where personal control fails. However, alarming a person specifically for these rare events based on physiology seems currently not viable as an ecological momentary intervention (EMI; Heron & Smyth (2010)), meaning outside the lab, since physiology and craving do not co-occur in sufficient frequencies over time outside the lab. Only for 57% of participants above average heart rate and craving co-occur in on average 31% of the events. This implies that 69% of the alarms will be false positives, which might become too burdensome (Beckjord & Shiffman, 2014) and disrupting (Yu et al., 2018), and participants could end up ignoring them (Nahum-Shani et al., 2018). However, this is under the assumption that all HR not accompanied by craving are false positives and not missed craving related incidents by the individual. As Baker et al. (2004) hypothesized, an individual might only experience craving if it surpasses a certain awareness threshold. Therefore prior to using wearables as EMI for specific biocueing in addiction treatment, future studies should explore what the true rate of false positives is of physiology and craving or lapses and whether that is an acceptable rate for biocueing (ter Harmsel et al., 2020). However, the specificity is high for participants, indicating not many craving incidents were missed by above average heartrate. Therefore, we do see the value of exploring the potential added benefit of using general, non-specific biocueing during treatment in the sense that many of the craving incidents happen between counseling sessions and due to recall bias patients forget what happened during their week. Furthermore, physiology could have a relation with relapse without a causal role of craving, meaning that physiology instead of craving is predictive of lapsing. Neutral reminders during the week of above average HR occurrences as implemented in many modern wearables or dedicated clinical apps (Derks et al., 2019) can support patients to recognize potential risky moments and help start the conversation about possible high risk situations during that week. This would also give the counselor an overall view of the client’s state, since both human and automated feedback gives the highest treatment effect (Wang & Miller, 2020).

### 5.5 Limitations

#### 5.5.1 Compliance rate

The compliance rate for self reported questions of on average 66% is comparable to the finding of 70% of Jones et al. (2018). Stone and Shiffman (2002) warn for the representativeness of the sampling when the non-response is 20% or higher, especially when the data is expected to be not missing at random. A case of not missing at random might be that participants stopped answering experience sampling questions or wearing the wearable technology when coming near to or during (re)lapses. There is a moderate positive correlation between compliance and relapse, in that the more an participant (re)lapsed the higher the compliance. Therefore it is not plausible that a lot of data was missing not at random. Additionally, the nonresponse Stone and Shiffman (2002) were warning for is mostly based on short EMA studies, where representativeness is a bigger issue.

#### 5.5.2 Study duration

EMA studies differ in duration, Serre et al. (2015) found that EMA studies in substance abuse are on average 34 days, with a max of 175. However, longer studies had often a less intensive design. Stone et al. (1991) recommended to use a max study duration of 2–4 weeks, due to a decline of data quality after these weeks. Van Berkel et al. (2019) found that the accuracy results for the working memory task did not change over the study duration, the accuracy of the recall task dropped as the study progressed. We expect to have little impact of this decline in recall accuracy, since the study mainly questioned current rather than past experiences. However, this study needed more weeks of data than the advised 2 to 4, due to the within person design and since (re)lapses were expected in less individuals in this first period (Kirshenbaum et al., 2009).

#### 5.5.3 E4

Another limitation of the study is the limited validity of the wearable device for EDA for smaller stressors (Menghini et al., 2019, van Lier et al., 2020). The low concordance between self-reported EDA and craving could be explained by the fact that the E4 wearable is not sensitive enough for precise EDA measurements. In a validation study the E4 wearable was found only to be valid for strong sustained stressors with TA and for HR also for smaller environmental stressors (van Lier et al., 2020). The low co-occurrence between TA and craving could be explained by the fact that craving is not a strong sustained stressor and more precise measures of TA are needed. Menghini et al. (2019) found no correlation between the golden standard (fingers) and the E4 (wrist) and hypothesized that the differences in measures could be due to the differences in sites. Hence, EDA measured at the fingers responds differently to emotional and cognitive stressors than EDA measured at the wrist. Menghini et al. (2019) also found the best results for HR, both on a cognitive and environmental stressor.

#### 5.5.4 Repeated single subject design

This was a repeated single subject or n-of-1 design (Vieira et al., 2017), hence with a small sample. Given that this study was the first of its kind, our focus was more on exploring the temporal and within-person fluctuations of craving (Drummond et al., 2000), rather than testing specific hypotheses. However, n-of-1 studies are more difficult to generalize to the population of interest. Vieira et al. (2017) argue that these studies are of particular interest when developing more tailored interventions. They state that if multiple studies explore the same topics with small samples, aggregation of multiple n-of-1 studies are possible with *meta*-analysis or mixed methods. We therefore recommend future studies to keep exploring the relations addressed in this study following the trailblazing approach we presented, in order to perform a *meta*-analysis of the larger participant base.

### 5.6 Conclusions

The current study is one of the first longitudinal EMA studies that investigated the association between craving and physiology in a within subject design in the daily life of recovering alcoholics. It is an important step towards the development of the use of wearable devices in alcohol treatment on the basis of physiological data, specifically measures related to cardiovascular fluctuations. This study underscores the importance of individual differences amongst people, as suggested by Drummond, 2000, Kavanagh et al., 2013. There is a real need for personalized research, maybe even individualized models and treatment (Alayan et al., 2018).

**Funding**: This work was supported by the University of Twente’s Tech4people program of the BMS faculty

## Declaration of Competing Interest

The authors declare that they have no known competing financial interests or personal relationships that could have appeared to influence the work reported in this paper.

## Figures and Tables

**Figure:**
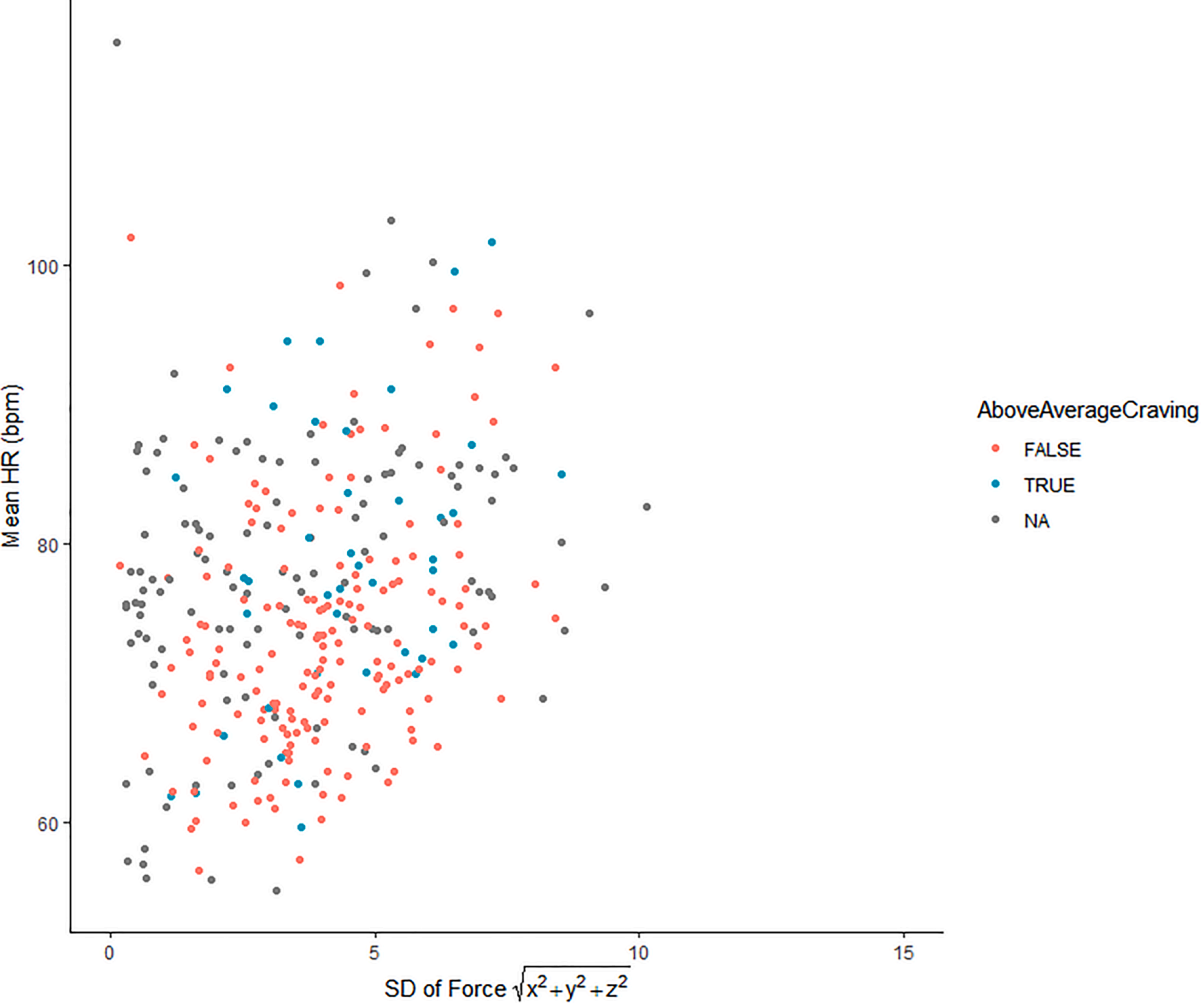


**Figure:**
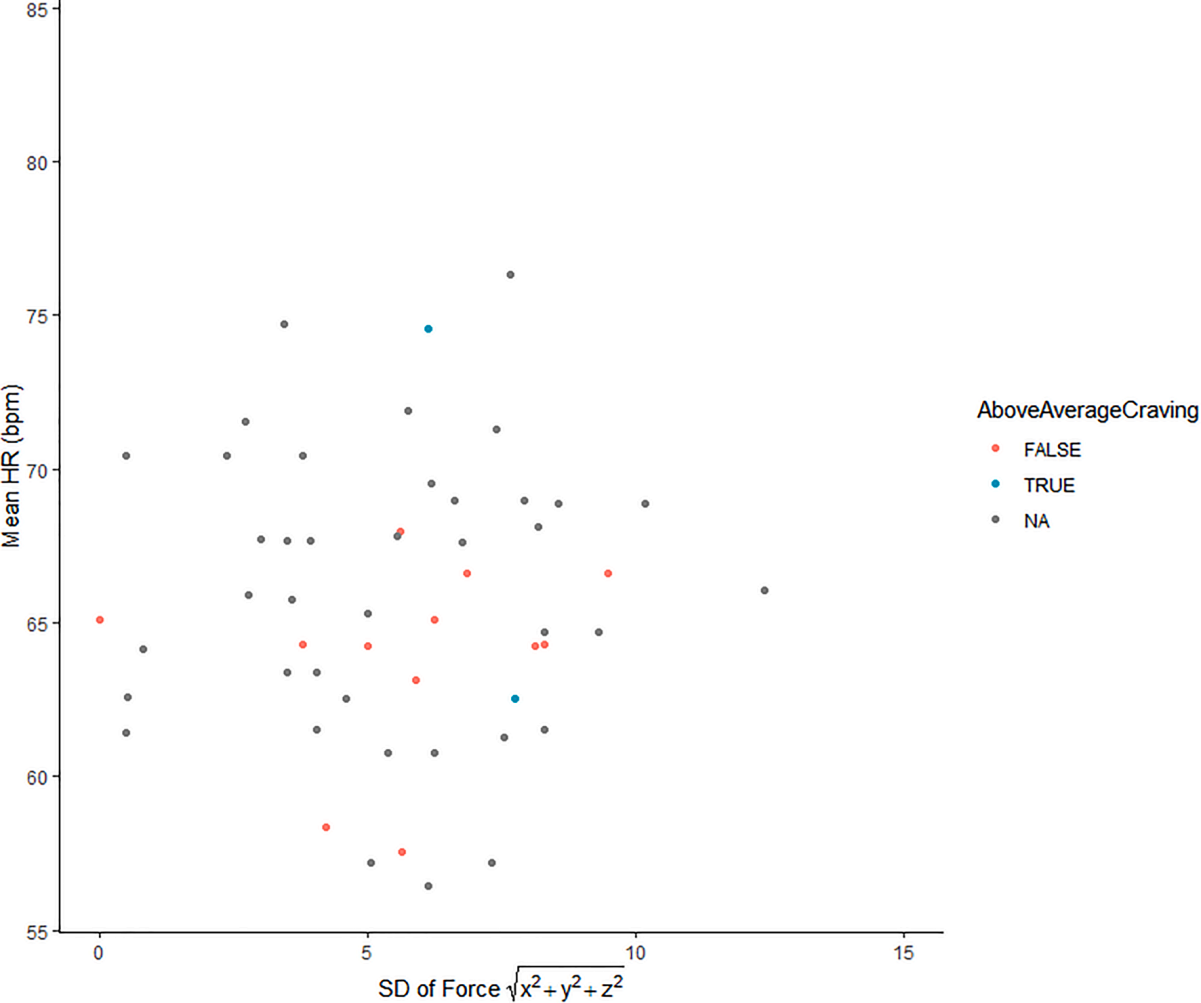


**Figure:**
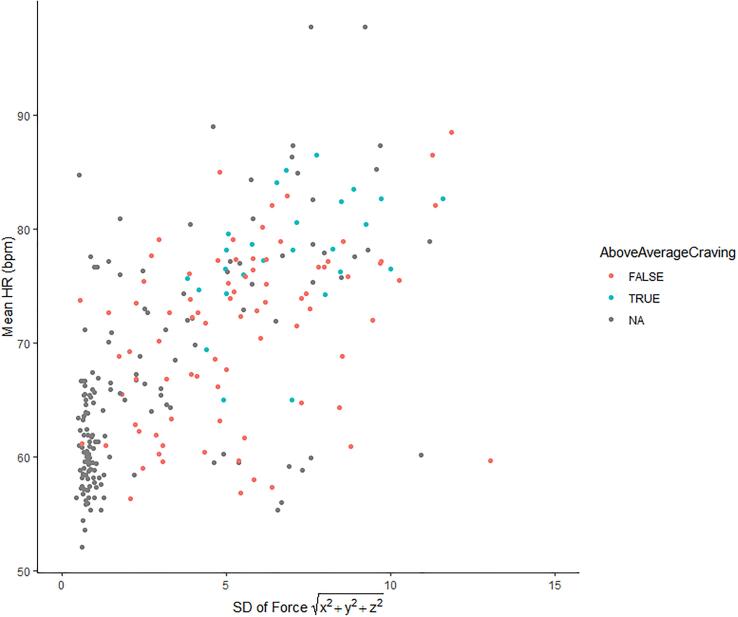


**Figure:**
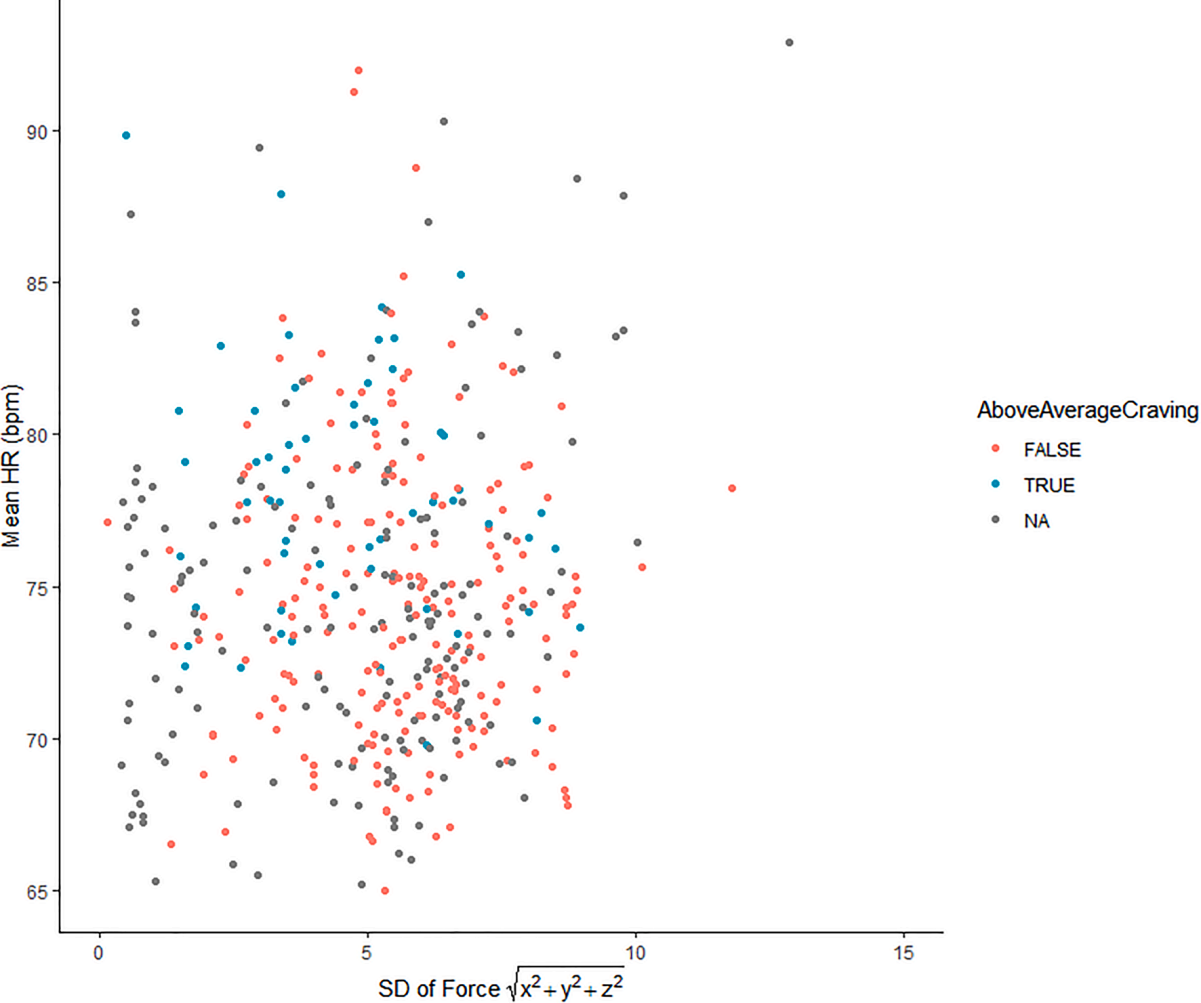


**Figure:**
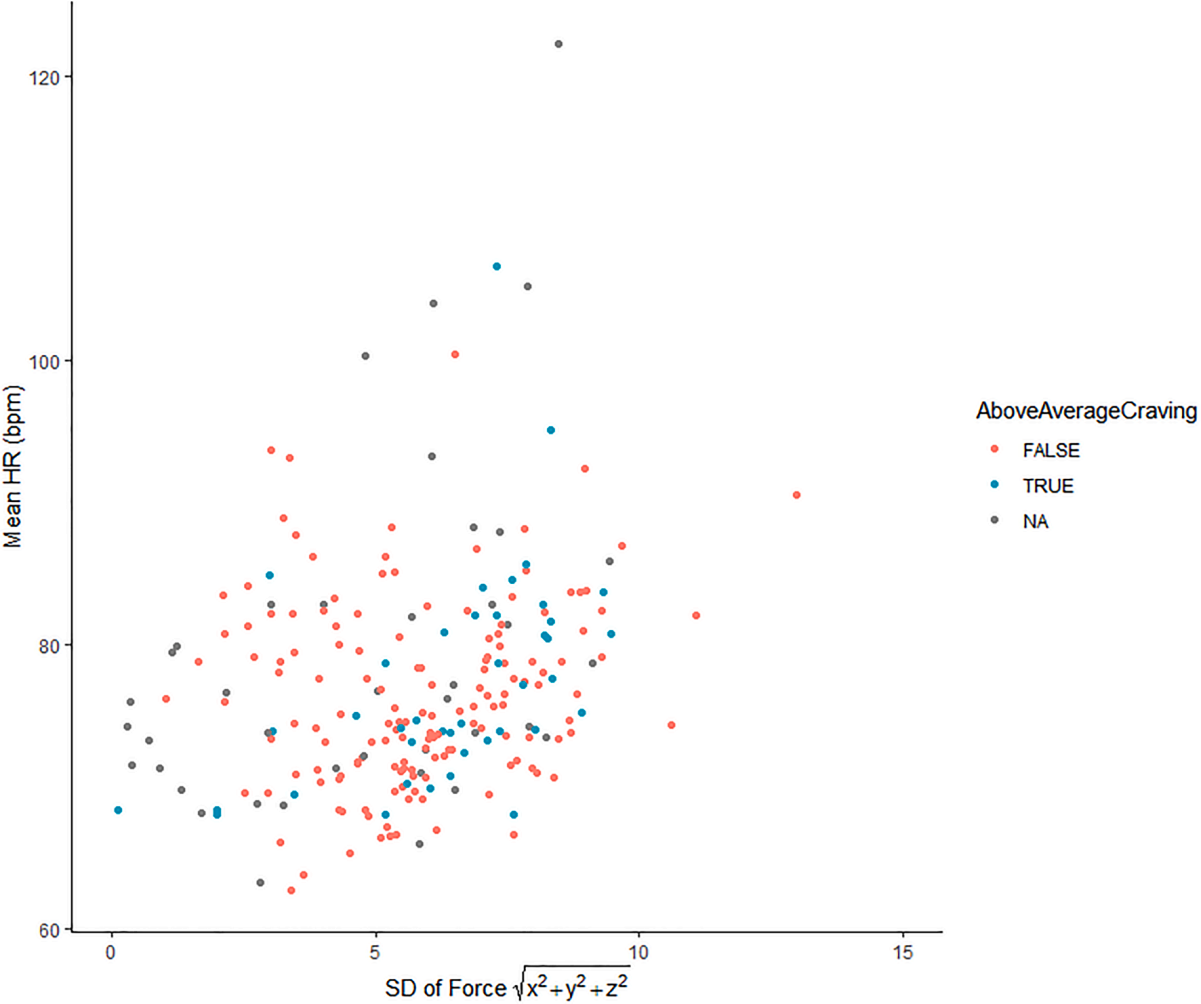


**Figure:**
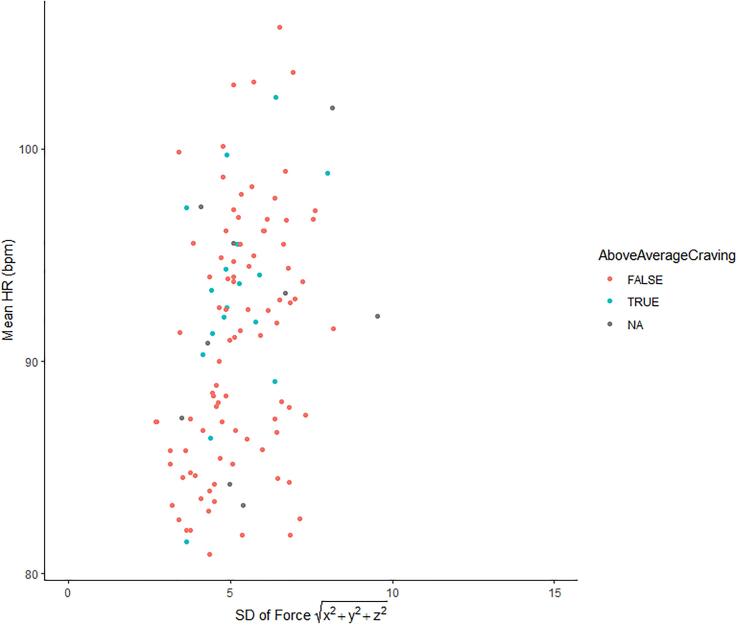


**Figure:**
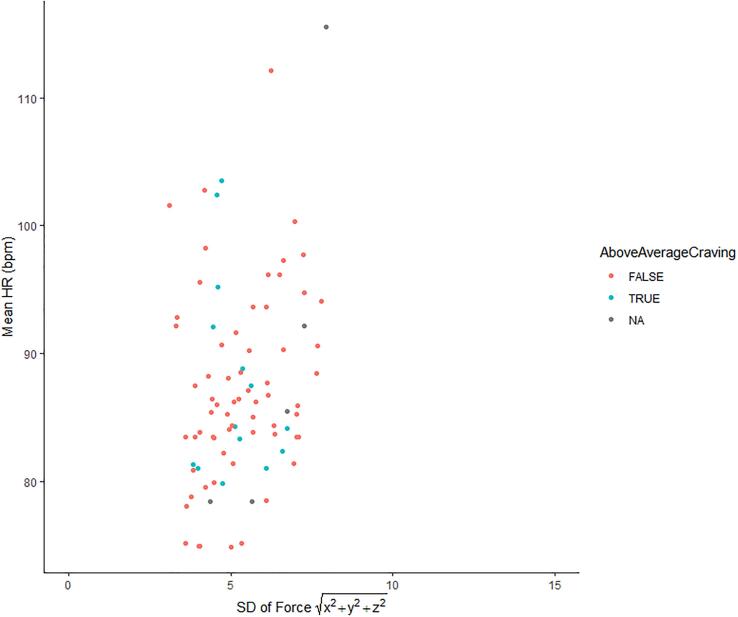


**Figure:**
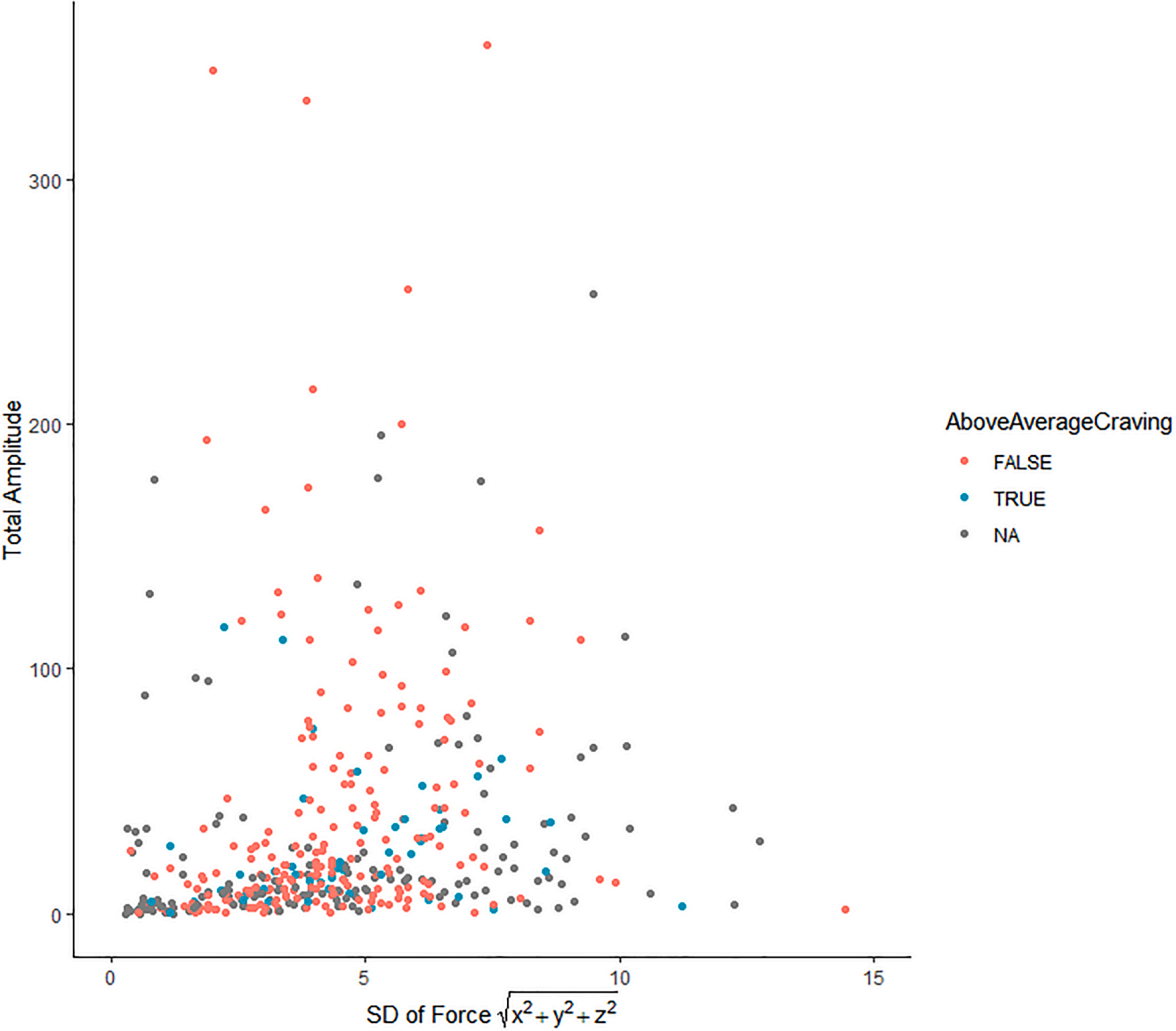


**Figure:**
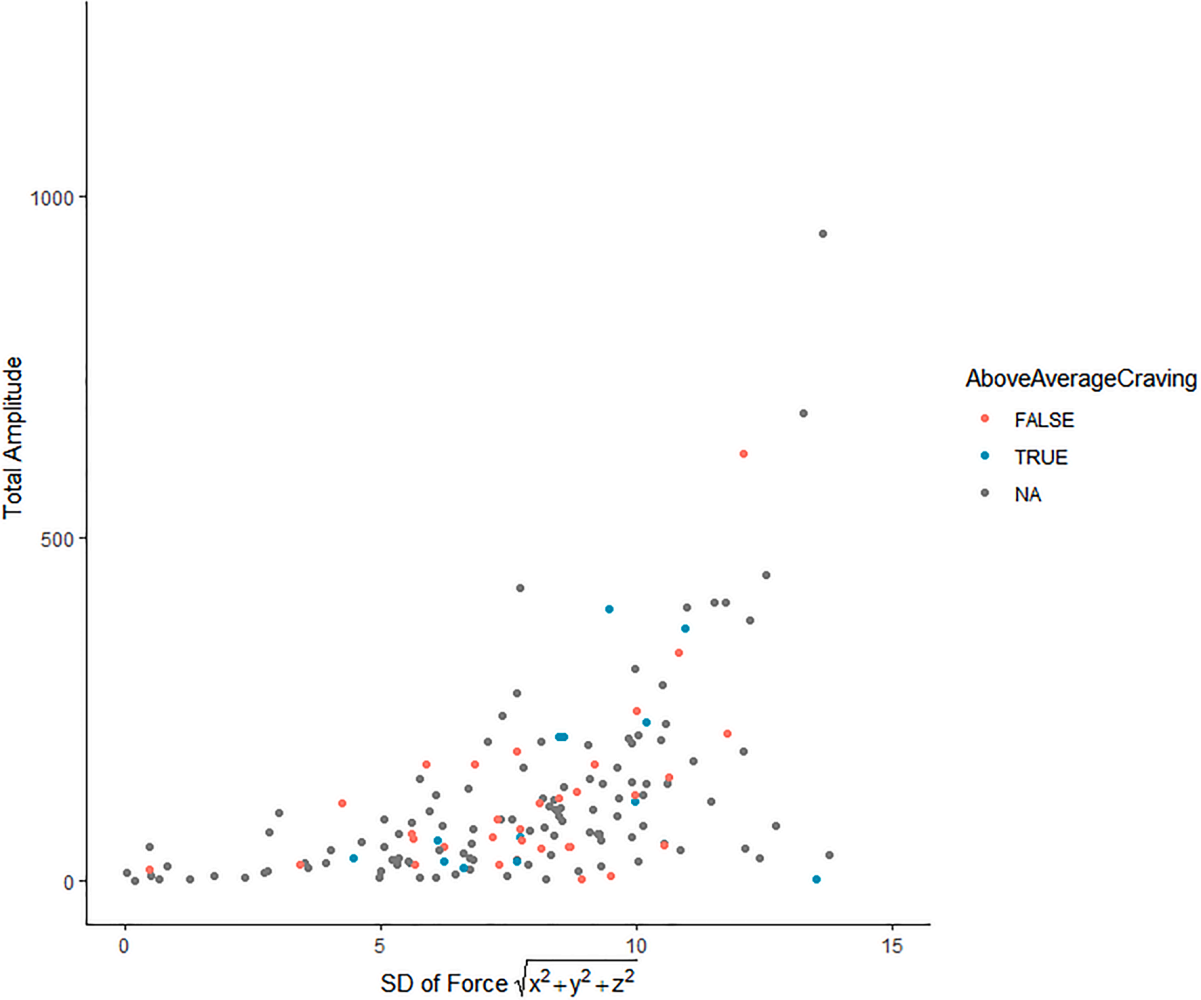


**Figure:**
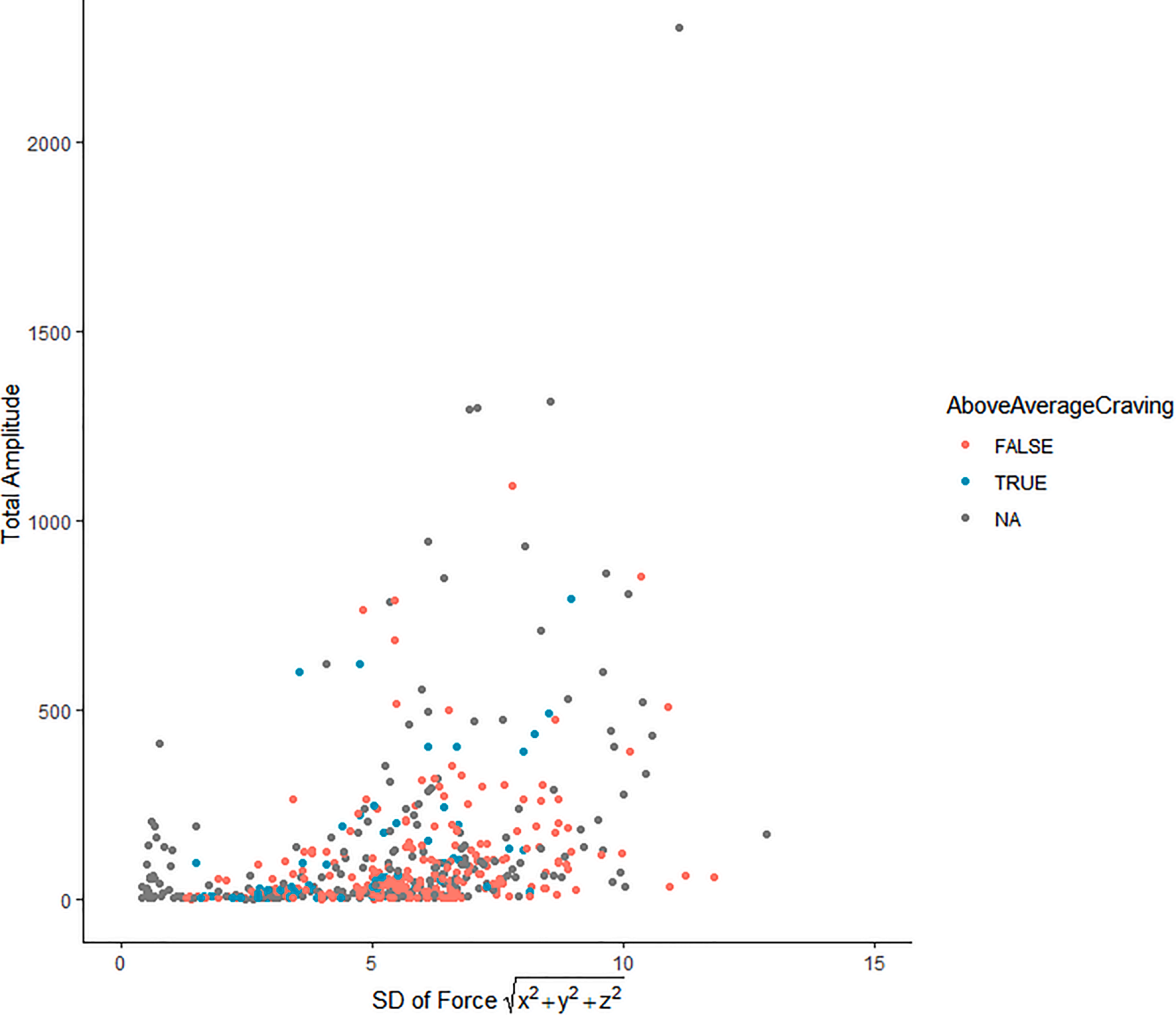


**Figure:**
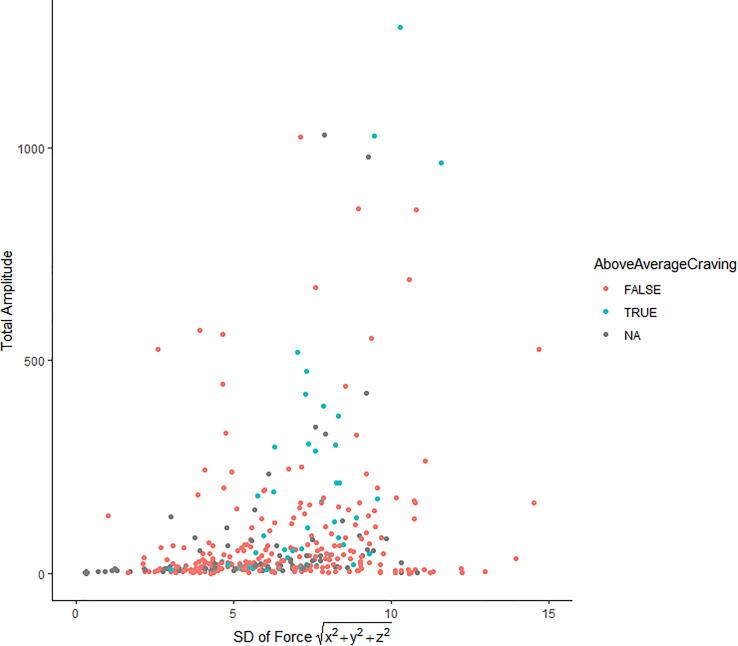


**Figure:**
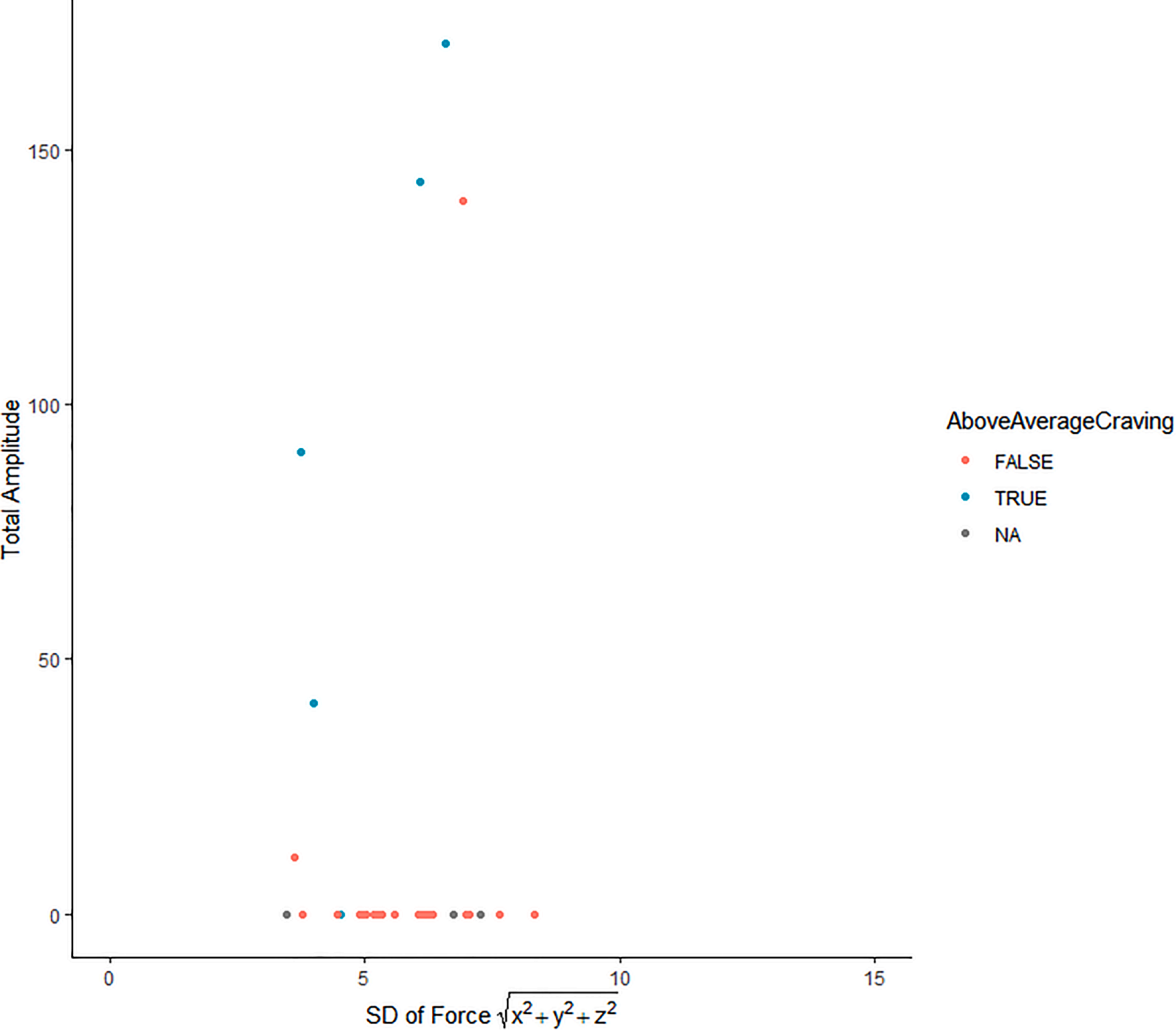


**Figure:**
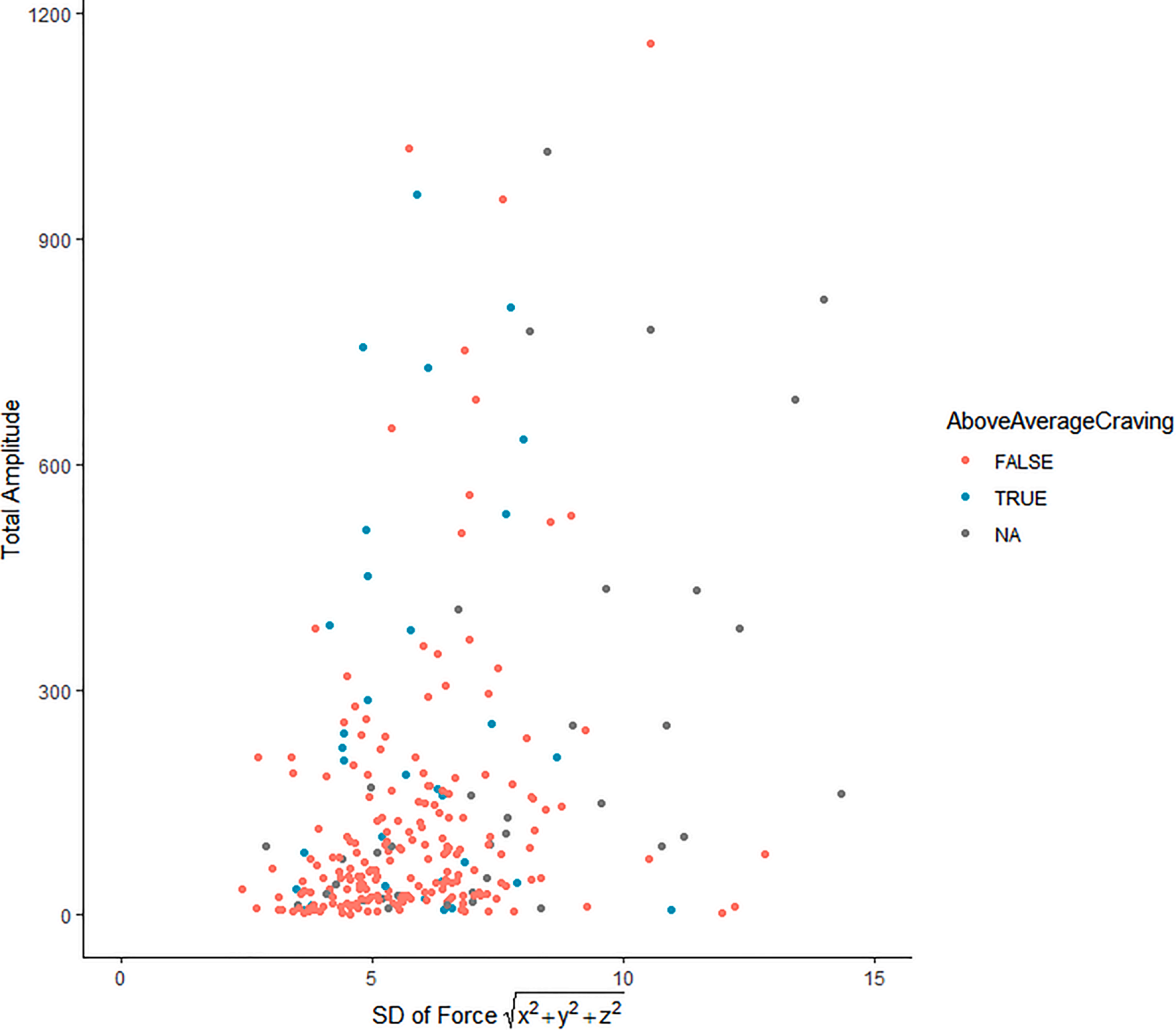


## Appendix A

Inclusion criteria

In order to be eligible to participate in this study, a participant had to meet all of the following criteria:

1. Older than 18.
2. Is moderate or severely use disorder according to the DSM-5.
3. Is currently or willing to be enrolled in the alcholdebaas online or face-to-face treatment phase.
4. Has aim of abstinence or drinking less. In case of drinking less, this aim has to include at least two days a week of not drinking at all.
5. Should be in possession of a personal mobile phone.

Exclusion criteria

A potential subject who meets any of the following criteria will be excluded from participation in this study:

1. Diagnosed with a psychiatric disease, suicidality, schizophrenia (known from the alcoholdebaas intake questionnaire) or Panic disorder
2. Multiple user (except nicotine, known from the alcholdebaas intake questionnaire)
3. Epilepsy
4. Cardiovascular or pulmonary disease
5. Pregnancy

## Appendix B

Impact of movement on mean HR and craving

In order to assess whether a correction of movement should be included, movement was measured with the wearable and the association with mean HR was inspected. If certain outliers of mean HR, meaning above a certain threshold of movement, were strongly related to only movement and consistently not with craving, these outliers are to be removed. The association between the SD of Force for every measurement moment (3 h average) was plotted against mean HR and above average Craving incidents were highlighted. The For participants 3 the correlation between mean HR and SD of Force is moderate, for others this relation is weak. However, the above craving incidents are randomly distributed over movement and not only found by lower SD of Force, showing that including a threshold of movement in order to heighten the sensitivity of mean HR to Craving is not justified.

Relation between HR, SD of Force and Craving for participant 1 (correlation = 0.25).

Relation between HR, SD of Force and Craving for participant 2 (correlation = 0.04).

Relation between HR, SD of Force and Craving for participant 3 (correlation = 0.62).

Relation between HR, SD of Force and Craving for participant 5 (correlation = 0.04).

Relation between HR, SD of Force and Craving for participant 6(correlation = 0.23).

Relation between HR, SD of Force and Craving for participant 9 (correlation = 0.36).

Relation between HR, SD of Force and Craving for participant 10 (correlation = 0.24).

## Appendix C

Impact of movement on Total Am and craving

In order to assess whether a correction of movement should be included, movement was measured with the wearable and the association with Total Amplitude was inspected. If certain outliers of Total Amplitude, meaning above a certain threshold of movement, were strongly related to only movement and consistently not with craving, these outliers are to be removed. The association between the SD of Force for every measurement moment (3 h average) was plotted against Total Amplitude and above average Craving incidents were highlighted. The For participants 3 the correlation between mean HR and SD of Force is moderate, for others this relation is weak. However, the above craving incidents are randomly distributed over movement and not only found by lower SD of Force, showing that including a threshold of movement in order to heighten the sensitivity of Total Amplitude to Craving is not justified.

Relation between Total Amplitude, SD of Force and Craving for participant 1 (correlation = 0.19).

Relation between Total Amplitude, SD of Force and Craving for participant 2 (correlation = 0.49).

Relation between Total Amplitude, SD of Force and Craving for participant 5 (correlation = 0.34).

Relation between Total Amplitude, SD of Force and Craving for participant 6 (correlation = 0.34).

Relation between Total Amplitude, SD of Force and Craving for participant 9 (correlation = 0.33).

Relation between Total Amplitude, SD of Force and Craving for participant 10 (correlation = 0.07).

## References

[b0015] Alayan N., Eller L., Bates M.E., Carmody D.P. (2018). Current evidence on heart rate variability biofeedback as a complementary anticraving intervention. Journal of Alternative and Complementary Medicine.

[b0020] Appelhans B.M., Luecken L.J. (2006). Heart rate variability as an index of regulated emotional responding. Review of General Psychology.

[b0025] Baker T.B., Piper M.E., McCarthy D.E., Majeskie M.R., Fiore M.C. (2004). Addiction motivation reformulated: An affective processing model of negative reinforcement. Psychological Review.

[b0030] Beckjord E., Shiffman S. (2014). Background for real-time monitoring and intervention related to alcohol use. Alcohol Research: Current Reviews.

[b0035] Benedek M., Kaernbach C. (2010). Decomposition of skin conductance data by means of nonnegative deconvolution. Psychophysiology.

[b0040] Bolger N., Laurenceau J.-P. (2013).

[b0045] Boucsein W., Fowles D.C., Grimnes S., Ben-Shakhar G., Roth W.T., Dawson M.E., Filion D.L. (2012). Publication recommendations for electrodermal measurements. Psychophysiology.

[b9015] Braithwaite J.J., Watson D.G., Jones R., Rowe M. (2013). A guide for analysing electrodermal activity (EDA) & skin conductance responses (SCRs) for psychological experiments. Psychophysiology.

[b0050] Cacioppo J.T., Tassinary L.G., Berntson G.G. (2016). Strong inference in psychophysiological science. Handbook of Psychophysiology, Fourth Edition.

[b0055] Carter B.L., Tiffany S.T. (1999). Meta-analysis of cue-reactivity in addiction research. Addiction.

[b0060] Chicco D. (2017). Ten quick tips for machine learning in computational biology. BioData Mining.

[b0065] Collins R.L., Morsheimer E.T., Shiffman S., Paty J.a., Gnys M., Papandonatos G.D. (1998). Ecological momentary assessment in a behavioral drinking moderation training program. Experimental and Clinical Psychopharmacology.

[b9005] Compton W.M., Cottler L.B., Dorsey K.B., Spitznagel E.L., Magera D.E. (1996). Comparing assessments of DSM-IV substance dependence disorders using CIDI-SAM and SCAN. Drug and alcohol dependence.

[b0070] Cooney N.L., Litt M.D., Morse P.a., Bauer L.O., Gaupp L. (1997). Alcohol cue reactivity, negative-mood reactivity, and relapse in treated alcoholic men. Journal of Abnormal Psychology.

[b0075] Cooney, Ned L, Litt, M. D., Cooney, J. L., & Pilkey, D. T. (2009). *NIH Public Access*. *21*(3), 277–286. https://doi.org/10.1037/0893-164X.21.3.277.Alcohol.

[b0080] Cooper M.L., Russell M., Skinner J.B., Windle M. (1992). Development and validation of a three-dimensional measure of drinking motives. Psychological Assessment.

[b0085] Cox W.M., Klinger E. (1988). A motivational model of alcohol use. Journal of Abnormal Psychology.

[b0090] DeMartini, K. S., Pittman, B., Krystal, J. H., O’Malley, S. S., & Krishnan-Sarin, S. (2020). Examining the relationship between self-reported drinking and in-laboratory drinking and craving: is there concordance? *Alcoholism: Clinical and Experimental Research*. 10.1111/acer.14329 PMC10158572 32352581

[b0095] Deng X., Liu Q., Deng Y., Mahadevan S. (2016). An improved method to construct basic probability assignment based on the confusion matrix for classification problem. Information Sciences.

[b0100] Derks Y.P.M.J., Klaassen R., Westerhof G.J., Bohlmeijer E.T., Noordzij M.L. (2019). Development of an ambulatory biofeedback app to enhance emotional awareness in patients with borderline personality disorder: Multicycle usability testing study. JMIR MHealth and UHealth.

[b0105] Drummond D.C. (2000). What does cue-reactivity have to offer clinical research?. Addiction.

[b0110] Drummond D.C. (2001). Theories of drugs craving, ancient and modern. Addiction.

[b0115] Drummond, D. C., Litten, R. Z., Lowman, C., & Hunt, W. A. (2000). Craving research: future directions. *Addiction*, *95*(8s2), 247–255. 10.1080/09652140050111816 11002919

[b0120] Enewoldsen N.M., Noordzij M.L., Pieterse M.E., van Lier H.G. (2016).

[b0125] Evers C., Hopp H., Gross J.J., Fischer A.H., Manstead A.S.R., Mauss I.B. (2014). Emotion response coherence: A dual-process perspective. Biological Psychology.

[b0130] Fisher A.J., Medaglia J.D., Jeronimus B.F. (2018). Lack of group-to-individual generalizability is a threat to human subjects research. Proceedings of the National Academy of Sciences.

[b0135] Ganganwar V. (2012). An overview of classification algorithms for imbalanced datasets. International Journal of Emerging Technology and Advanced Engineering.

[b0140] Helzer J.E., Badger G.J., Searles J.S., Rose G.L., Mongeon J.A. (2006). Stress and alcohol consumption in heavily drinking men: 2 Years of daily data using interactive voice response. Alcoholism: Clinical and Experimental Research.

[b0145] Heron K.E., Smyth J.M. (2010). Ecological momentary interventions: Incorporating mobile technology into psychosocial and health behaviour treatments. British Journal of Health Psychology.

[b0150] Higley A.E., Crane N.A., Spadoni A.D., Quello S.B., Goodell V., Mason B.J. (2011). Craving in response to stress induction in a human laboratory paradigm predicts treatment outcome in alcohol-dependent individuals. Psychopharmacology (Berl).

[b0155] Holt L.J., Litt M.D., Cooney N.L. (2012). Prospective analysis of early lapse to drinking and smoking among individuals in concurrent alcohol and tobacco treatment. Psychology of Addictive Behaviors : Journal of the Society of Psychologists in Addictive Behaviors.

[b9010] Intille, S. S. (2012). Emerging technology for studying daily life.

[b0160] Jones A., Remmerswaal D., Verveer I., Robinson E., Franken I.H.A., Wen C.K.F., Field M. (2018). Compliance with ecological momentary assessment protocols in substance users: A meta-analysis. Addiction.

[b0165] Kavanagh D.J., Statham D.J., Feeney G.F.X., Young R.M.D., May J., Andrade J., Connor J.P. (2013). Measurement of alcohol craving. Addictive Behaviors.

[b0170] Kirshenbaum A.P., Olsen D.M., Bickel W.K. (2009). A quantitative review of the ubiquitous relapse curve. Journal of Substance Abuse Treatment.

[b0175] Krahn D.D., Bohn M.J., Henk H.J., Grossman J.L., Gosnell B. (2005). Patterns of urges during early abstinence in alcohol-dependent subjects. The American Journal on Addictions / American Academy of Psychiatrists in Alcoholism and Addictions.

[b0180] Kuerbis A.N., Shao S., Treloar Padovano H., Jadanova A., Selva Kumar D., Vitale R., Morgenstern J. (2020). Context and craving among individuals with alcohol use disorder attempting to moderate their drinking. Experimental and Clinical Psychopharmacology.

[b0185] Kuppens P., Oravecz Z., Tuerlinckx F. (2010). Feelings change: Accounting for individual differences in the temporal dynamics of affect. Journal of Personality and Social Psychology.

[b0190] Lalkhen A.G., McCluskey A. (2008). Clinical tests: Sensitivity and specificity. Continuing Education in Anaesthesia Critical Care & Pain.

[b0195] Larimer M.E., Palmer R.S., Marlatt G.A. (1999). Relapse prevention: An overview of Marlatt’s cognitive-cehavioral model. Alcohol Research & Health.

[b0200] Litt M.D., Kadden R.M., Kabela-Cormier E. (2009). Individualized assessment and treatment program for alcohol dependence: Results of an initial study to train coping skills. Addiction.

[b0205] Luque A., Carrasco A., Martín A., de las Heras A. (2019). The impact of class imbalance in classification performance metrics based on the binary confusion matrix. Pattern Recognition.

[b0210] Maisto S.A., Witkiewitz K., Moskal D., Wilson A.D. (2016). Is the construct of relapse heuristic, and does it advance alcohol use disorder clinical practice?. Journal of Studies on Alcohol and Drugs.

[b0215] Marôco, J. (2018). *Análise Estatística com o SPSS Statistics.: 7ª edição*. ReportNumber, Lda.

[b0220] Martinotti G., Di Nicola M., Tedeschi D., Callea A., Di Giannantonio M., Janiri L. (2013). Craving Typology Questionnaire (CTQ): A scale for alcohol craving in normal controls and alcoholics. Comprehensive Psychiatry.

[b0225] Mehl M.R., Conner T.S. (2012). Getting started: Launching a study in daily life. Handbook of Research Methods for Studying Daily Life.

[b0230] Menghini L., Gianfranchi E., Cellini N., Patron E., Tagliabue M., Sarlo M. (2019). Stressing the accuracy: Wrist-worn wearable sensor validation over different conditions. Psychophysiology.

[b0235] Miller W.R., Westerberg V.S., Harris R.J., Tonigan J.S. (1996). What predicts relapse? Prospective testing of antecedent models. Addiction.

[b0240] Miranda R., Ray L., Blanchard A., Reynolds E.K., Monti P.M., Chun T., Ramirez J. (2014). Effects of naltrexone on adolescent alcohol cue reactivity and sensitivity: An initial randomized trial. Addiction Biology.

[b0245] Moskowitz D.S., Russell J.J., Sadikaj G., Sutton R. (2009). Measuring people intensively. Canadian Psychology/Psychologie Canadienne.

[b0250] Musthag, M., Raij, A., Ganesan, D., Kumar, S., & Shiffman, S. (2011). Exploring micro-incentive strategies for participant compensation in high-burden studies. *Proceedings of the 13th International Conference on Ubiquitous Computing - UbiComp ’11*, 435. https://doi.org/10.1145/2030112.2030170.

[b0255] Nahum-Shani I., Smith S.N., Spring B.J., Collins L.M., Witkiewitz K., Tewari A., Murphy S.A. (2018). Just-in-time adaptive interventions (JITAIs) in mobile health: Key components and design principles for ongoing health behavior support. Annals of Behavioral Medicine.

[b0260] Ooteman W., Koeter M.W.J., Vserheul R., Schippers G.M., Brink W. (2006). Measuring craving: an attempt to connect subjective craving with cue reactivity. Alcoholism: Clinical and Experimental Research.

[b9035] Quintana D.S., Guastella A.J., McGregor I.S., Hickie I.B., Kemp A.H. (2013). Heart rate variability predicts alcohol craving in alcohol dependent outpatients: Further evidence for HRV as a psychophysiological marker of self-regulation. Drug and Alcohol Dependence.

[b0265] Ray L. (2013). Natural Environment.

[b0270] Reis, H. T. (2012). Why researchers should think “real-world”: A conceptual rationale. In *Handbook of research methods for studying daily life* (pp. 3–21). http://www.redi-bw.de/db/ebsco.php/search.ebscohost.com/login.aspx?direct=true&db=psyh&AN=2012-05165-001&site=ehost-live.

[b0275] Reynolds, E. K., & Monti, P. M. (2013). *The cue reactivity paradigm in addiction research*.

[b0280] Robinson T.E., Berridge K.C. (1993). The neural basis of drug craving: An incentive-sensitization theory of addiction. Brain Research Reviews.

[b0285] Rohsenow D.J., Monti P.M. (1999). Does urge to drink predict relapse after treatment?. Alcohol Research & Health : The Journal of the National Institute on Alcohol Abuse and Alcoholism.

[b0290] Rosenberg H. (2009). Clinical and laboratory assessment of the subjective experience of drug craving. Clinical Psychology Review.

[b0295] Sardá-Espinosa, A., Subbiaha, S., & Bartz-Beielstein, T. (2017). *Conditional Inference Trees for the Knowledge Extraction from Motor Health Condition Data*.

[b0300] Serre, F., Fatseas, M., Swendsen, J., & Auriacombe, M. (2015). Ecological momentary assessment in the investigation of craving and substance use in daily life: A systematic review. In *Drug and Alcohol Dependence* (Vol. 148, pp. 1–20). Elsevier Ireland Ltd. https://doi.org/10.1016/j.drugalcdep.2014.12.024. 10.1016/j.drugalcdep.2014.12.024 25637078

[b0305] Shiffman, S. (2009). *Ecological Momentary Assessment (EMA) in Studies of Substance Use*. *21*(4), 486–497. https://doi.org/10.1037/a0017074. 10.1037/a0017074 PMC2846437 19947783

[b0310] Shiffman S., Paty J.A., Gnys M., Kassel J.A., Hickcox M. (1996). First lapses to smoking: Within-subjects analysis of real-time reports. Journal of Consulting and Clinical Psychology.

[b9030] Shiffman S., Stone A.A., Hufford M.R. (2008). Ecological momentary assessment. Annu. Rev. Clin. Psychol..

[b0315] Shoda Y., LeeTiernan S. (2002).

[b0320] Skinner M.D., Aubin H.J. (2010). Craving’s place in addiction theory: Contributions of the major models. Neuroscience and Biobehavioral Reviews.

[b0325] Stone A.A., Kessler R.C., Haythomthwatte J.A. (1991). Measuring Daily Events and Experiences: Decisions for the Researcher. Journal of Personality.

[b0330] Stone A.A., Shiffman S. (2002). Capturing momentary, self-report data: A proposal for reporting guidelines. Annals of Behavioral Medicine.

[b0335] Swift A., Heale R., Twycross A. (2020). What are sensitivity and specificity?. Evidence-Based Nursing.

[b0340] Taylor J. (2004). Electrodermal reactivity and its association to substance use disorders. Psychophysiology.

[b0345] Taylor, S., Jaques, N., Weixuan Chen, Fedor, S., Sano, A., & Picard, R. (2015). Automatic identification of artifacts in electrodermal activity data. *2015 37th Annual International Conference of the IEEE Engineering in Medicine and Biology Society (EMBC)*, 1934–1937. https://doi.org/10.1109/EMBC.2015.7318762. 10.1109/EMBC.2015.7318762 PMC5413200 26736662

[b0350] ter Harmsel J.F., Noordzij M.L., Goudriaan A.E., Dekker J.J.M., Swinkels L.T.A., van der Pol T.M., Popma A. (2020). Biocueing and ambulatory biofeedback to enhance emotion regulation: A review of studies investigating non-psychiatric and psychiatric populations. International Journal of Psychophysiology..

[b0355] Tidey J.W., Monti P.M., Rohsenow D.J., Gwaltney C.J., Miranda R., McGeary J.E., Paty J.A. (2008). Moderators of naltrexone’s effects on drinking, urge, and alcohol effects in non-treatment-seeking heavy drinkers in the natural environment. Alcoholism: Clinical and Experimental Research.

[b9000] Tiffany S.T. (1999). Cognitive concepts of craving. Alcohol Research & Health.

[b0360] Tiffany S.T., Conklin C.A. (2000). A cognitive processing model of alcohol craving and compulsive alcohol use. Addiction.

[b0365] Van Berkel N., Ferreira D., Kostakos V. (2017). The experience sampling method on mobile devices. ACM Computing Surveys (CSUR).

[b0370] Van Berkel N., Goncalves J., Koval P., Hosio S., Dingler T., Ferreira D., Kostakos V. (2019). Context-informed scheduling and analysis: Improving accuracy of mobile self-reports. Conference on Human Factors in Computing Systems - Proceedings.

[b0375] van Lier H.G., Pieterse M.E., Garde A., Postel M.G., de Haan H.A., Vollenbroek-Hutten M.M.R., Noordzij M.L. (2020). A standardized validity assessment protocol for physiological signals from wearable technology: Methodological underpinnings and an application to the E4 biosensor. Behavior Research Methods.

[b9025] van Lier H.G., Oberhagemann M., Stroes J.D., Enewoldsen N.M., Pieterse M.E., Schraagen J.M.C., Noordzij M.L. (2017). April). Design decisions for a real time, alcohol craving study using physio-and psychological measures.

[b0380] van Lier H.G., Pieterse M.E., Schraagen J.M.C., Postel M.G., Vollenbroek-Hutten M.M.R., de Haan H.A., Noordzij M.L. (2018). Identifying viable theoretical frameworks with essential parameters for real-time and real world alcohol craving research: A systematic review of craving models. Addiction Research & Theory.

[b0385] Verheul R., van den Brink W., Geerlings P. (1999). A three-pathway psychobiological model of craving for alcohol. Alcohol and Alcoholism.

[b0390] Vieira R., McDonald S., Araújo-Soares V., Sniehotta F.F., Henderson R. (2017). Dynamic modelling of n-of-1 data: Powerful and flexible data analytics applied to individualised studies. Health Psychology Review.

[b0395] Wang L., Miller L.C. (2020). Just-in-the-moment adaptive interventions (JITAI): A meta-analytical review. Health Communication.

[b0400] Wang Q., Li W., Liu X.S., Carroll J.S., Jänne O.A., Krasnickas E., Brown M. (2014). NIH Public Access..

[b0405] Waters, A. J., Schoenmakers, T. M., Snelleman, M., Szeto, E. H., Franken, I. H. A., Hendriks, V. M., & van de Mheen, D. (2020). Affect, motivation, temptation, and drinking among alcohol-dependent outpatients trying to maintain abstinence: An Ecological Momentary Assessment study. *Drug and Alcohol Dependence*, *206*(August 2019), 107626. https://doi.org/10.1016/j.drugalcdep.2019.107626. 10.1016/j.drugalcdep.2019.107626 31786398

[b0410] Witteman J., Post H., Tarvainen M., De Bruijn A., Perna E.D.S.F., Ramaekers J.G., Wiers R.W. (2015). Cue reactivity and its relation to craving and relapse in alcohol dependence: A combined laboratory and field study. Psychopharmacology (Berl).

[b0415] Wray T.B., Merrill J.E., Monti P.M. (2014). Using ecological momentary assessment (EMA) to assess situation-level predictors of alcohol use and alcohol-related consequences. Alcohol Research : Current Reviews.

[b0420] Yu B., Funk M., Hu J., Wang Q., Feijs L. (2018). Biofeedback for everyday stress management: A systematic review. Frontiers in ICT.

[b0425] Zheng Y., Cleveland H.H., Molenaar P.C.M., Harris K.S. (2015). An alternative framework to investigating and understanding intraindividual processes in substance abuse recovery: an idiographic approach and demonstration. Evaluation Review.

